# Exosome biopotentiated hydrogel restores damaged skeletal muscle in a porcine model of stress urinary incontinence

**DOI:** 10.1038/s41536-022-00240-9

**Published:** 2022-09-29

**Authors:** Tyler J. Rolland, Timothy E. Peterson, Raman Deep Singh, Skylar A. Rizzo, Soulmaz Boroumand, Ao Shi, Tyra A. Witt, Mary Nagel, Cassandra K. Kisby, Sungjo Park, Lois A. Rowe, Christopher R. Paradise, Laura R. E. Becher, Brooke D. Paradise, Paul G. Stalboerger, Emanuel C. Trabuco, Atta Behfar

**Affiliations:** 1 Van Cleve Cardiac Regenerative Medicine Program, Mayo Clinic Center for Regenerative Medicine, Rochester, MN USA; 2 Mayo Clinic Department of Cardiovascular Medicine, Rochester, MN USA; 3 Mayo Clinic Medical Sciences Training Program, Rochester, MN USA; 4 Mayo Clinic Department of Molecular Pharmacology and Experimental Therapeutics, Rochester, MN USA; 5 Mayo Clinic Division of Urogynecology, Rochester, MN USA; 6 Marriott Heart Disease Research Program, Mayo Clinic, Rochester, MN USA; 7 Rion LLC, Rochester, MN US

**Keywords:** Regenerative medicine, Translational research

## Abstract

Urinary incontinence afflicts up to 40% of adult women in the United States. Stress urinary incontinence (SUI) accounts for approximately one-third of these cases, precipitating ~200,000 surgical procedures annually. Continence is maintained through the interplay of sub-urethral support and urethral sphincter coaptation, particularly during activities that increase intra-abdominal pressure. Currently, surgical correction of SUI focuses on the re-establishment of sub-urethral support. However, mesh-based repairs are associated with foreign body reactions and poor localized tissue healing, which leads to mesh exposure, prompting the pursuit of technologies that restore external urethral sphincter function and limit surgical risk. The present work utilizes a human platelet-derived CD41a and CD9 expressing extracellular vesicle product (PEP) enriched for NF-κB and PD-L1 and derived to ensure the preservation of lipid bilayer for enhanced stability and compatibility with hydrogel-based sustained delivery approaches. In vitro, the application of PEP to skeletal muscle satellite cells in vitro drove proliferation and differentiation in an NF-κB-dependent fashion, with full inhibition of impact on exposure to resveratrol. PEP biopotentiation of collagen-1 and fibrin glue hydrogel achieved sustained exosome release at 37 °C, creating an ultrastructural “bead on a string” pattern on scanning electron microscopy. Initial testing in a rodent model of latissimus dorsi injury documented activation of skeletal muscle proliferation of healing. In a porcine model of stress urinary incontinence, delivery of PEP-biopotentiated collagen-1 induced functional restoration of the external urethral sphincter. The histological evaluation found that sustained PEP release was associated with new skeletal muscle formation and polarization of local macrophages towards the regenerative M2 phenotype. The results provided herein serve as the first description of PEP-based biopotentiation of hydrogels implemented to restore skeletal muscle function and may serve as a promising approach for the nonsurgical management of SUI.

## Introduction

Urinary incontinence is a common condition that afflicts between 10 and 40% of women in the United States^1^. Stress urinary incontinence (SUI), the involuntary loss of urine with exertional physical activity, accounts for approximately one-third of cases and leads to ~200,000 yearly surgical interventions^2,3^. Although not life-threatening, urinary incontinence greatly impacts a woman’s quality of life; with similar Health Utility Index scores being reported among women seeking treatment for SUI and community-dwelling women with other chronic, debilitating illnesses^4^.

The interplay of sub-urethral support and urethral sphincter contraction is typically required to achieve optimal coaptation and maintain continence during activities that result in increased intra-abdominal pressure^2,3^. Events such as vaginal delivery lead to a disruption of these coordinated events and result in continence compromise. Most surgical approaches to correct SUI have focused on re-establishing sub-urethral support with minimal attention paid to restoring urethral sphincter function^5,6^. Since its invention in the late 1990’s, the minimally invasive mid-urethral sling, a procedure that utilizes a narrow strip of polypropylene mesh placed sub-urethral, has grown to account for ~90% of the yearly incontinence surgeries owing to its high success and low complications^7^. Due to fears about the use of mesh for prolapse reconstructive surgery following 2008 and 2011 FDA public health warnings, the mid-urethral sling has been decreasing in popularity^8,9^. A population-based cohort utilizing a robust national database of inpatient surgical procedures showed that the number of SUI surgical interventions performed in Scotland decreased from ~830 in 2005–2006 to ~180 in 2015–2016, respectively^10^. Similar trends have been observed in our practice at Mayo Clinic in Rochester MN; with the number of sling, procedures performed each year decreasing by over 50% between 2009 and 2016^11^. As the prevalence of SUI is not expected to have changed during this time interval, these data suggest that women have limited their willingness to undergo mesh-based surgical correction of SUI and highlights a critical need to provide safer and effective nonsurgical and non-mesh-based treatment options.

Cell-based approaches have led to the experimental use of stem cells at various locations along the urinary tract to drive myocyte repopulation in preclinical models. Renewed interest in the satellite cell after its discovery in the nineteenth century resulted in its incorporation into SUI biologics^12–18^. Animal and pilot human studies have implemented muscle precursor cells (MPC) to reconstitute the injured external urethral sphincter and improve continence. In these experiments, MPCs are harvested from the quadriceps femoris, enriched in the laboratory, and injected in the mid-urethra (approximate position of the sphincter muscle). However, MPC harvest is morbid and cellular expansion is time-consuming and costly, making this traditional cell-based regenerative approach to SUI unlikely to achieve widespread application^19^. The ongoing investigation is being conducted to determine whether homogenous autologous satellite cell cultures or heterogeneous cellular material is more effective^18,20^. Neither allogenic nor variations of autologous therapy have been successful in permanently attenuating SUI at the time this paper was written. Despite the significant impact of SUI on women’s health, the approaches continue to be utilized in SUI cases due to procedural discomfort, infection, high cost, lifestyle limitations, and cumbersome self-care routines.

Successful skeletal muscle regeneration requires a concert of molecular events which encompass NF-κB p65 signaling^21–26^, in tandem with the recruitment of immune effectors to drive skeletal muscle healing^27^. Recent works have indicated upregulation of MyoD1, Myf5, and Pax7 in the early treatment of skeletal muscle (SKM) injury is associated with upregulation of NF-κB in regenerating tissue^26^. Furthermore, NF-κB has long been established as a critical signaling factor in the perpetuation of myoblast proliferation in tandem with SKM differentiation^28,29^. In parallel, the presence of the M2 macrophage population appears to be essential for skeletal muscle homeostasis following exercise^27,30^, as well as in SKM regenerative events^31^. Interestingly, macrophage polarization towards the regenerative M2 fate has been shown to be driven through an SDF-1 and PD-L1 dependent pathway^25,32–37^. Cell-free approaches, including gene therapy, recombinant growth factors, and exosomes, have been considered for skeletal muscle regeneration; however, the short duration of effect has limited therapeutic impact.

Platelet activation is known to trigger a series of events that culminate in the release of exosomes from the platelet multivesicular body (MVB). Integrin-α2B or CD41 is a classic marker for platelets indicative of the megakaryocytic lineage and has been utilized to track platelet exosome biogenesis when co-expressed with tetraspanins such as CD9 and CD63^38–40^. In this effort, an apheresis human platelet population was maintained within a suspension culture, facilitating the collection of a platelet extracellular vesicle population (PEP) enriched for CD41 and the exosomal marker CD9, expressing NF-κB and PD-L1, reflecting key established targets for SKM regeneration. Extracellular vesicle purification was performed to ensure full lipid bilayer integrity for maximal preservation of exosome biopotency at ambient and body temperature. In vitro application of exosomes to muscle myoblasts induced chemotaxis, proliferation, and differentiation of this population in a NF-ΚB-dependent fashion. In vivo, sustained delivery of exosomes within a biopotentiated hydrogel demonstrated SKM repair in a rat model of volumetric muscle loss. In a porcine SUI model, minimally invasive delivery of a PEP collagen-1 hydrogel resulted in functional restoration of the external sphincter associated with augmented muscle proliferation, local upregulation of NF-ΚB and PD-L1, and polarization of local macrophages towards the M2 fate. The work presented herein provides mechanistic and translational insight for the use of an acellular biologic able to induce local stem cell migration and differentiation following minimally invasive delivery in a porcine SUI model.

## Results

### Exosome characterization

**Fig. 1:**
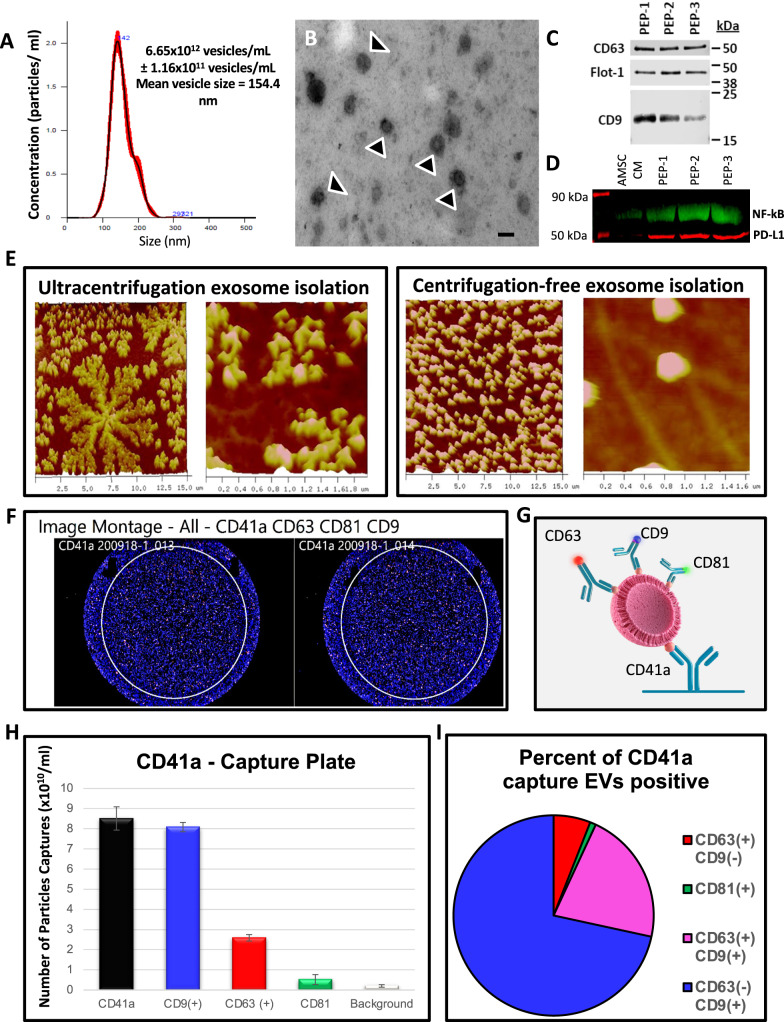
Purified exosome product (PEP) characterization, quantification, and delivery biodistribution. **A** NanoSight nanoparticle analysis of size distribution and concentration of PEP diluted in phosphate buffered saline 1:1000 documented 6.65 × 10^12^ ± 1.16 × 10^11^. **B** Transmission electron microscopy of PEP. Scale = 200 nm arrow heads pointing to EVs. **C** Western blot probing for CD63, CD9, and Flotillin-1 in 3 separate CGMP manufactured PEP lots. **D** Western blot comparison of NF-κB p65 and PD-L1 levels, in 3 separate CGMP manufactured PEP lots versus adipose-derived mesenchymal stem cell conditioned media (AMSC-CM). **E** Atomic-force microscope comparing platelet-conditioned medium EV isolation using centrifugation versus the PEP process, scale bar embedded in the image. **F** Representative image from Single-particle interferometric reflectance imaging sensing (SP-IRIS) analysis for presence of surface CD41a, CD9, CD63, and CD81 tetraspanins. **G** Graphical representation of the SP-IRIS analysis. **H** Quantitation of CD9, CD63 and CD81 on a CD41a captured plate documented vast majority of PEP as CD41a/CD9 positive, with smaller representation from CD63 and background CD81. Data presented as mean ± stdev. *N* = 3 separate CGMP manufactured PEP lots. **I** Pie chart representation of the exosome tetraspanin surface marker profile of CD41a captured PEP exosomes.

Extracellular vesicles engineered from platelet suspension cultures were assessed for size consistency, high degree of lipid bilayer integrity, and markers conformant with exosome populations^41,42^ (Fig. 1). NanoSight characterization of PEP revealed particle concentration of ~6.65 × 10^12^ particles/mL and size mean of ~154.4 nm (Fig. 1A and Supplementary Fig. 1). Electron microscopy of PEP solution confirm NanoSight vesicle size distribution; with most of the purified vesicles ~100–150 nm (Fig. 1B). Western blot analysis demonstrated consistent expression of CD63, CD9 and Flotillin-1, established exosomal markers, in 3 separate clinical preparations of PEP (Fig. 1C). PEP preparations were documented to be enriched for NF-κB p65 (2.41× fold; *p* < 0.05; Student’s *t*-test) and PD-L1 (4.98× fold; *p* < 0.005; Student’s *t*-test) compared to the levels seen in extracellular vesicles (EVs) purified from adipose-derived mesenchymal stem cells conditioned media (AMSC-CM; Fig. 1D). Atomic-force microscopy of EVs purified from platelets using ultracentrifugation document disruption of the phospholipid bilayer, which was preserved with avoidance of high sheer stress purification (Fig. 1E). To confirm derived EVs are platelet exosome preparations (PEP), there were evaluated for presence of concomitant Integrin-α2b (CD41) and CD9 expression using single-particle interferometric reflectance imaging sensing (SP-IRIS) analysis of relative quantity of CD63 (6.67 ± 0.58%), CD9 (76.0 ± 0.0%), CD81 (1.0 ± 0.0%), and CD9/CD63 (16.67 ± 0.58%) within CD41a-positive exosomes (Fig. 1F–I). Real-time microscopy tracked PEP trafficking with documentation of HUVEC uptake and localization into the PKH26 labeled PEP within the endosomal compartment (Supplementary Fig. 2 and Supplementary Movie 1). DiR labeled PEP exhibited localization to the site of intramuscular injection on in vivo monitoring (Supplementary Fig. 3) with blinded GLP histological evaluation showing no on- or off-target pathological events (Supplementary Fig. 4 and Supplementary Table 1).

### Human skeletal muscle myoblast (HSMM) co-cultured with PEP shows dose-dependent growth, chemotaxis, cell migration, and skeletal muscle differentiation

**Fig. 2:**
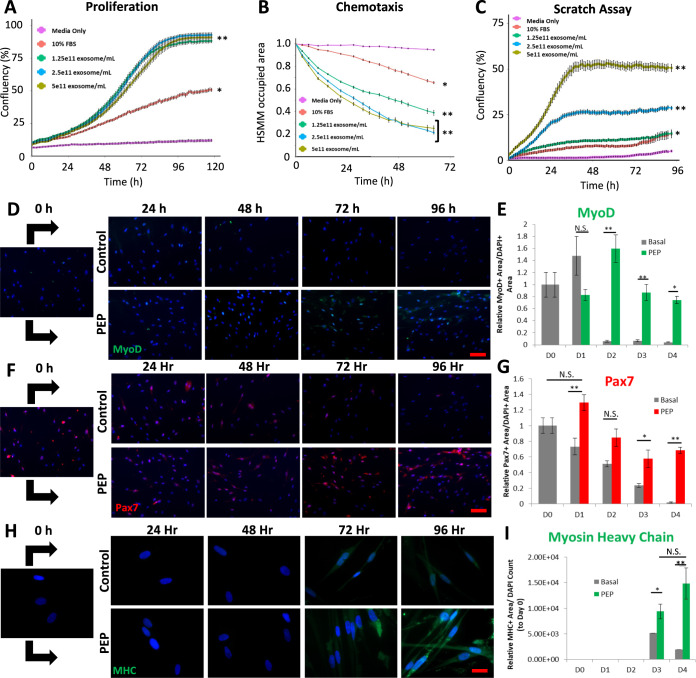
Purified exosome product functional characterization and in vitro human skeletal muscle myoblasts (HSMM) culturing with increasing concentration of PEP. **A** IncuCyte proliferation, **B** chemotaxis, and **C** wound scratch analyses of HSMM grown with increasing concentration of PEP ranging from 1.25 × 10^11^ exosomes/mL to 5 × 10^11^ exosomes/mL and media supplemented with 10% fetal bovine serum (FBS) versus serum-free media. **D**–**I** Representative immunocytochemistry of a 96 h time-course of HSMM cultured with serum-free media (Control) or serum-free media plus 2.5 × 10^11^ exosomes/mL PEP (PEP). Immunostaining for MyoD (**D**; green, scale = 100 µm), Pax7 (**F**; red, scale = 100 µm), and Myosin Heavy Chain (MHC, **H**; green, scale = 20 µm). Nuclei counterstained with DAPI (blue). ImageJ blind quantification of **E** MyoD+ Area/DAPI + Area, **G** Pax7+ Area/DAPI + Area, and **I** Myosin Heavy Chain+ Area/DAPI object count, relative to day zero. N.S. Not significant; *p < 0.05; **p < 0.005. Data represents AVE ± SEM. Student’s *t*-test was utilized.

At each concentration tested, myoblasts grown with PEP achieved higher percent confluency compared to serum-free media and media supplemented with 10% fetal bovine serum (FBS) (Fig. 2A). At PEP concentrations higher than 1.25 × 10^11^ exosomes/ml myoblast grew to over 90% confluency compared to only ~50% confluency achieved with standard growth media with 10% FBS. Similarly, a dual chamber migration assay documented that PEP concentrations greater than 2.5 × 10^11^ exosomes/ml significantly decreased the total phase area in the top chamber, suggesting significant more myoblast migration through the pores and improved chemotaxis (Fig. 2B). This was validated in a scratch assay showing enhanced wound confluency in PEP treated (≥1.25 × 10^11^ exosomes/ml) conditions versus FBS (Fig. 2C). Myogenic lineage maturation from satellite cells (the endogenous skeletal muscle stem cell) to multinucleated myotubes (Fig. 2D–I) was documented with 2.5 × 10^11^ exosomes/ml of PEP. Specifically, the myogenic lineage from myoblasts (Pax7 + and MyoD + ) to myotubes (Myosin Heavy Chain + ) was identified after 96 h by culturing HSMM with PEP (Fig. 2D–I, Supplementary Figs. 5 and 6). By day 4, in contrast to basal media (1949 ± 1106, pixel intensity/area), PEP was able to induce significant MHC induction (14,859 ± 3049, pixel intensity/area, *p* < 0.01; Student’s *t*-test; Supplementary Table 2), to drive myogenic lineage maturation (Fig. 2H, I).

### Resveratrol inhibits PEP-mediated HSMM proliferation suggesting NF-κB as a central driver of satellite cell proliferation and differentiation

**Fig. 3:**
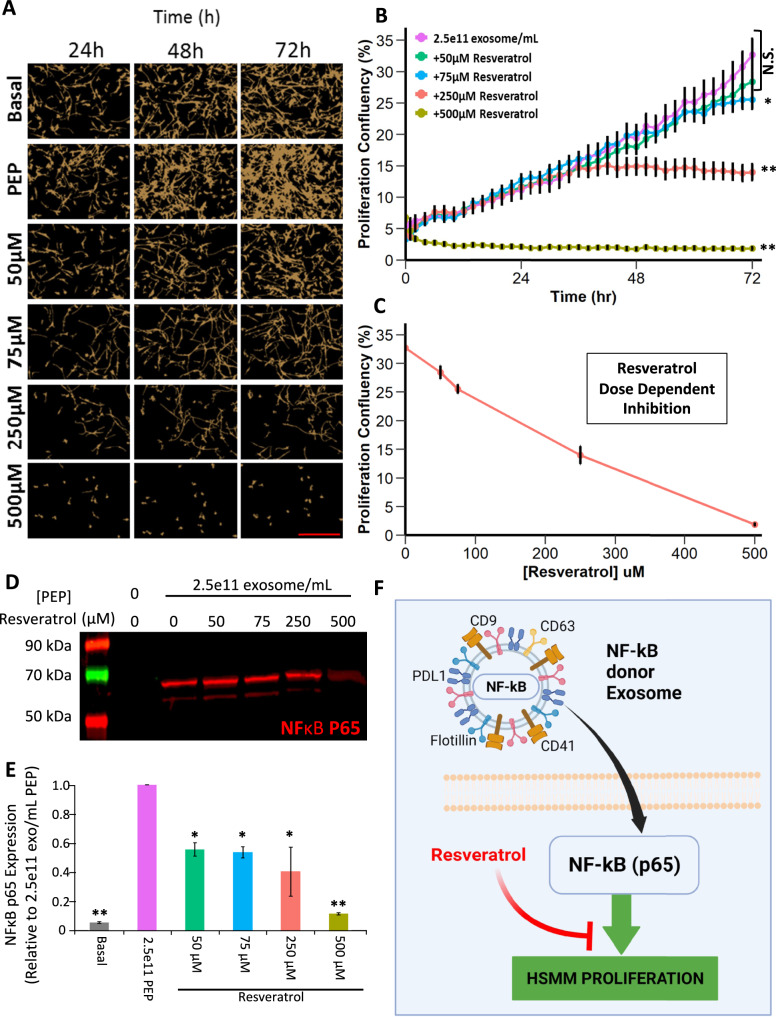
Resveratrol dose-dependent inhibition of PEP-mediated HSMM proliferation. **A** Representative live cell images and **B** proliferation growth curves of HSMM cultured with basal media, 2.5 × 10^11^ exosomes/mL PEP, and 2.5 × 10^11^ exosomes/mL PEP with increasing concentrations of resveratrol. Scale = 400 um. (N.S. not significant, *p < 0.05, **p < 0.0001). **C** Resveratrol dose-dependent inhibition of HSMM proliferation. Data points from corresponding live cell analysis of respective growth conditions (Fig. 2A, B) at t = 72 h. **D** Western blot of NF-κB p65 expression in HSMM cells cultured with media supplemented with 2.5 × 10^11^ exosomes/mL PEP alone or with increasing concentrations of resveratrol. **E** Basal NF-κB p65 protein expression compared to the expression obtained from cells cultured in 2.5 × 10^11^ exosomes/mL PEP alone or with increasing resveratrol concentrations (all comparisons to PEP, *p < 0.05, **p < 0.0001). **F** Schematic of hypothesized mechanism of PEP donation and role of NF-κB p65 on skeletal muscle proliferation. Data represents AVE ± SEM. Student’s *t*-test was utilized.

To assess whether PEP donation of NF-ΚB p65 was responsible for mediating myogenic proliferation and differentiation^28,29^, repeat HSMM proliferation, cell count, and morphology analysis with PEP alone or in combination with increasing concentrations of resveratrol (Fig. 3), a known inhibitor of NF-κB (p65) was performed^43–47^. Myoblast proliferation was inhibited in a dose-dependent fashion, with resveratrol at 75, 250, and 500 µM significantly inhibiting proliferation by ~20%, 50%, and 100%, respectively (PEP vs 75 µM = *p* < 0.05, PEP vs 250 µM = *p* < 0.0001, PEP vs 500 µM = *p* < 0.0001; Student’s *t*-test; Fig. 3A–C). Western blot analysis of cells grown in each condition showed a dose-dependent decrease in NF-ΚB expression following resveratrol treatment suggesting a role for this transcription factor in myoblast proliferation and differentiation (PEP vs negative = *p* < 0.0001, PEP vs 50 µM = *p* < 0.05, PEP vs 75 µM = *p* < 0.05, PEP vs 250 µM = *p* < 0.05, PEP vs 500 µM = *p* < 0.0001; Student’s *t*-test; Fig. 3D, E). These findings confirm PEP-driven increases in HSMM NF-κB p65 levels and the importance of this mitogenic factor in driving skeletal myoblast growth (Fig. 3F).

### Repair of rat volumetric muscle loss (VML) latissimus dorsi defect with purified exosome product enhances skeletal muscle regeneration

**Fig. 4:**
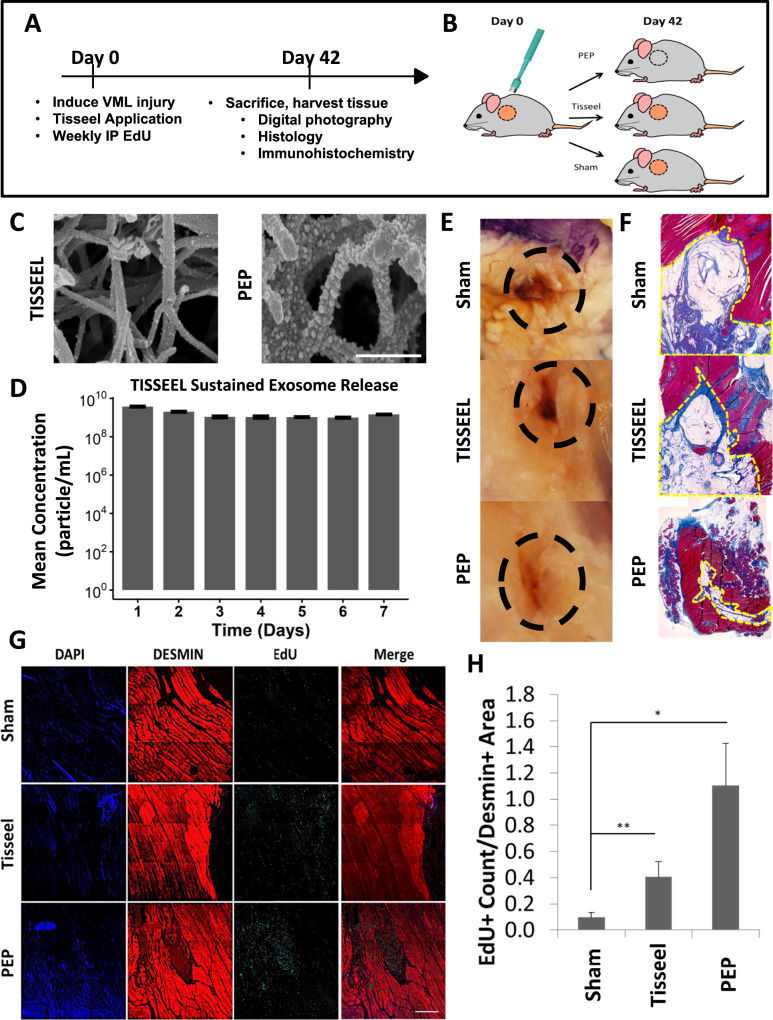
Repair of rat volumetric muscle loss latissimus dorsi defect with PEP. **A** Study timeline. **B** Schematic of latissimus dorsi volumetric muscle loss rat model. **C** Scanning electron microscopy of Tisseel (Left) and Tisseel reconstituted with 1 × 10^12^ exosomes/mL PEP (Right). Scale = 500 nm. **D** Tisseel release assay to show sustained exosome release. Triplicates are reported every 24 h and serum-free media was replaced daily. **E** Representative photographs of gross healing at injury site and **F** Masson’s Trichrome staining of VML sites treated with saline (top), Tisseel (middle), and Tisseel reconstituted with 1 × 10^12^ exosomes/mL PEP (Bottom). Dashed region highlights area of biopsy punch injury. Images obtained with ×10 magnification. **G** Representative sections of immunostaining of VML sites treated with saline sham (Top), Tisseel (Middle), and Tisseel reconstituted with 1 × 10^12^ exosomes/mL PEP (Bottom). Nuclei were counterstained with DAPI (blue), skeletal muscle protein Desmin (red), and cell proliferation with EdU (cyan). Scale Bar = 500 µm. **H** ImageJ blind quantification of de novo skeletal muscle proliferation by calculating the proportion of EdU-positive cells per mm^2^ of Desmin-positive area (red). *p < 0.05. **p < 0.005. Data represents AVE ± SEM. Student’s *t*-test was utilized.

To assess the impact of PEP on skeletal muscle regeneration in vivo, we utilized a rat volumetric muscle loss model, where a large muscle lesion was created to exceed the endogenous satellite stem cell capabilities for self-repair. Latissimus dorsi defects (8 mm) were created with a punch biopsy and treated with saline (sham; *n* = 9), clinical-grade fibrin glue (Tisseel, *n* = 9), or Tisseel reconstituted with 1 × 10^12^ exosomes/ml (PEP, *n* = 9) (Fig. 4A, B). Following reconstitution, PEP exosomes uniformly adhered to fibrin fibers as documented by scanning electron microscopy (Fig. 4C) to create a “bead on a string” pattern. Furthermore, Tisseel hydrogel biopotentiated with 1 × 10^12^ exosomes/ml resulted in the sustained release of exosomes (Fig. 4D and Supplementary Table 3). Animals were treated with 2’-Deoxy-5-ethynyluridine (EdU) as a thymidine analog to monitor cellular division and proliferation.

There we no deaths or infections prior to sacrifice at 8 weeks. Sham-treated animals had persistent muscle defects with fatty infiltration while those repaired with Tisseel or with Tisseel-PEP had grossly healed defects (Fig. 4E). Histological characterization revealed that in contrast to the absence of tissue in sham, Tisseel alone induced primarily a fatty infiltrate with the significant inflammatory response. Conversely, defects treated with PEP had widespread skeletal muscle regrowth with a resulting decrease in the defect compared to both sham and Tisseel-treated rats (Fig. 4E, F). Immunohistochemical evaluation of PEP treated cohorts revealed colocalization of EdU+ cells in Desmin+ tissue areas suggesting de novo skeletal muscle regrowth at the injury site (Fig. 4G); with significantly more EdU staining Desmin-positive cells per mm^2^ compared to sham control (*p* < 0.001; Student’s *t*-test), but not compared to Tisseel alone (*p* = 0.24; Student’s *t*-test) (Fig. 4H). Whereas the above data suggest PEP-biopotentiated Tisseel hydrogel impacts muscle regeneration at the site of injury, this model was not amenable to assessing skeletal muscle function.

### Restoration of urethral pressures following urethral sphincter repair with PEP in a novel porcine SUI model

**Fig. 5:**
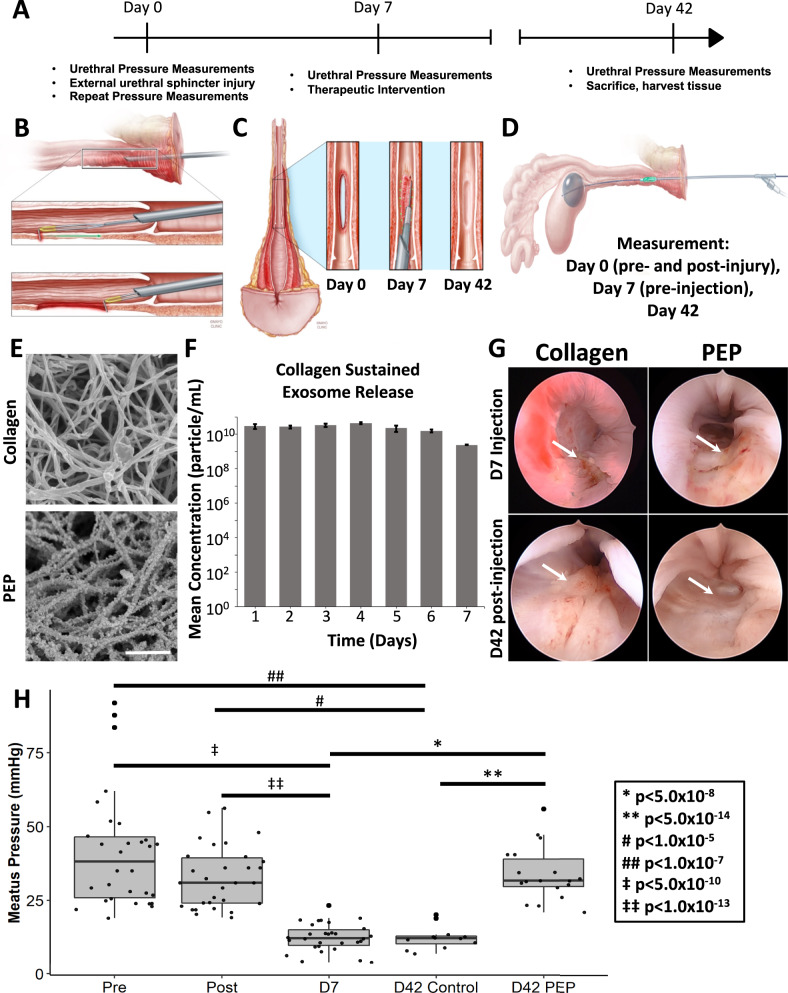
Creation of porcine SUI model and urethral pressures following repair of urethral sphincter lesion with PEP. **A** Study timeline. **B** Schematic of cystoscopic induced transurethral full thickness, focal urethral sphincter defect to create the porcine SUI model. Note the apposition of the urethral sphincter muscle to the anterior surface of the vagina and urethra (top image). **C** Schematic of gross appearance of the urethral sphincter defect at day 0, prior to injecting the intervention (Day 7), and prior to euthanasia (Day 42). **D** Modified Medspira mCompass pressure catheter highlighting stabilization of the catheter at the bladder neck (gray) and movable pressure sensor (green) used to obtain pressures along the length of the pig’s vagina and urethra. **E** Scanning electron microscopy of collagen (Top) and collagen reconstituted with 1 × 10^12^ exosomes/mL PEP (Bottom). Scale = 1 µm. **F** Collagen release assay to show sustained exosome release (*n* = 3). **G** Representative cystoscopic images of the urethral sphincter lesion on day 7 prior to delivering the intervention and on day 42 prior to euthanasia. Arrows point to the site of the urethral sphincter defect. **H** Graph of mean urethral pressures pre- and post-injury on day 0 (*n* = 10), pre-injection on day 7 (*n* = 10) and prior to euthanasia on day 42. Collagen = injection of collagen; PEP = injection of collagen reconstituted with 1 × 10^12^ exosomes/mL PEP. D42 Control = urethral sphincter defect treated with collagen (*n* = 4); Day 42 PEP = urethral sphincter defect treated with collagen reconstituted with 1 × 10^12^ exosomes/mL PEP (*n* = 6). Bar graph data represents AVE ± SEM, while box-plot data represents the IQR (gray box) and whiskers represent 1.5 IQR. One way ANOVA with post-hoc Tukey HSD was utilized.

To assess if PEP-mediated skeletal muscle regeneration can integrate with uninjured surrounding muscle to restore the pre-injury function of the urethral sphincter, we created a porcine model mimicking SUI. This model was chosen due to anatomic similarities between human and pig urethral sphincters. A total of 10 female Yorkshire-Crossed Pigs weighing 70–80 kg were utilized (4 controls and 6 experimental). Under aseptic conditions and general anesthesia, an ~2 cm long full thickness lesion (extending the urethral wall and overlying the urethral sphincter) was created cystoscopically at the 6 o’clock position starting 1 cm from the meatus and extending cephalad (Fig. 5A, B). The animals were allowed to convalesce for ~7 d and returned to the operating room where a total of 5 ml of collagen (collagen) or collagen with 1 × 10^12^ exosomes/ml (PEP) was injected in ~0.5 cc aliquots over 10 injection points along the length of the previously created urethral sphincter defect (Fig. 5C). A urethral pressure profile was utilized to quantify sphincter function using a modified Medspira mCompass manometry pressure catheter. Pressures were recorded pre- and post-injury creation on day 0, at day 7–10, and prior to euthanasia on day 42 post-therapy (Fig. 5D). Urethral pressures were recorded for 10 sec, starting in the vagina, and extending to the bladder, neck in 1 cm increments (Fig. 5D). In contrast to TISSEEL, which polymerizes instantly, 5 mg/ml collagen hydrogel takes ~78 s to gel at 37 °C, making it more suitable as the flowable hydrogel for injection through a cystoscope. PEP demonstrated a similar binding affinity to collagen as it did to TISSEEL on SEM (Fig. 5E). Additionally, collagen-1 hydrogel biopotentiated with 1 × 10^12^ exosomes/ml resulted in the sustained release of exosomes (Fig. 5F and Supplementary Table 3). Three pressure readings from each 10 s recorded segment were averaged to produce a mean urethral pressure at that location (Supplementary Fig. 7). EdU was administered throughout the study as a thymidine analog to monitor cellular division and proliferation.

There were neither deaths nor infections observed during the study. Two cases of urinary retention (1-Collagen, 1-PEP) were observed, and both resolved within 48 h. One animal, in the PEP treatment group, developed overt urinary incontinence following lesion creation and continued to have incontinence following PEP injection with documented reduction of incontinence (overt on days 1–9 post-treatment, exercise-induced by day 27, and no leakage by day 29). This was documented in the medical record suggesting this approach provides a model with the desired impact on urethral sphincter function, with reversal noted following PEP therapy. Urethral mucosa from both treatment groups was fully intact at 42 d cystoscope follow-up (Fig. 5G). Although the mean urethral pressure minimally changed on day 0 (41.3 ± 3.57 mmHg pre vs 32.74 ± 1.91 mmHg post-injury, *p* = 0.039 ANOVA with post-hoc Tukey HSD), it dropped significantly by day 7 (12.46 ± 0.85 mmHg; *p* < 0.0001; ANOVA with post-hoc Tukey HSD), quantitatively validating the model (Fig. 5H, Supplementary Tables 4–6, Supplementary Fig. 8). At day 42, treatment with collagen alone resulted in no improvement in sphincter function as measured by urethral pressure (12.37 ± 1.13 mmHg; *p* = 0.95; ANOVA with post-hoc Tukey HSD). In contrast, animals treated with PEP had significant restoration of urethral pressures (33.9 ± 2.15 mmHg; *p* < 0.0001; ANOVA with post-hoc Tukey HSD) versus day 7 pre-injection and day 42 collagen alone (Fig. 5H, Supplementary Tables 4–6, Supplementary Fig. 8). Similar findings were observed for animals treated cystoscopically, with the prototype injection device, and in the pooled analysis (Fig. 5H, Supplementary Fig. 8, Supplementary Tables 4–6).

### Histological characterization of urethral sphincter lesion repair

**Fig. 6:**
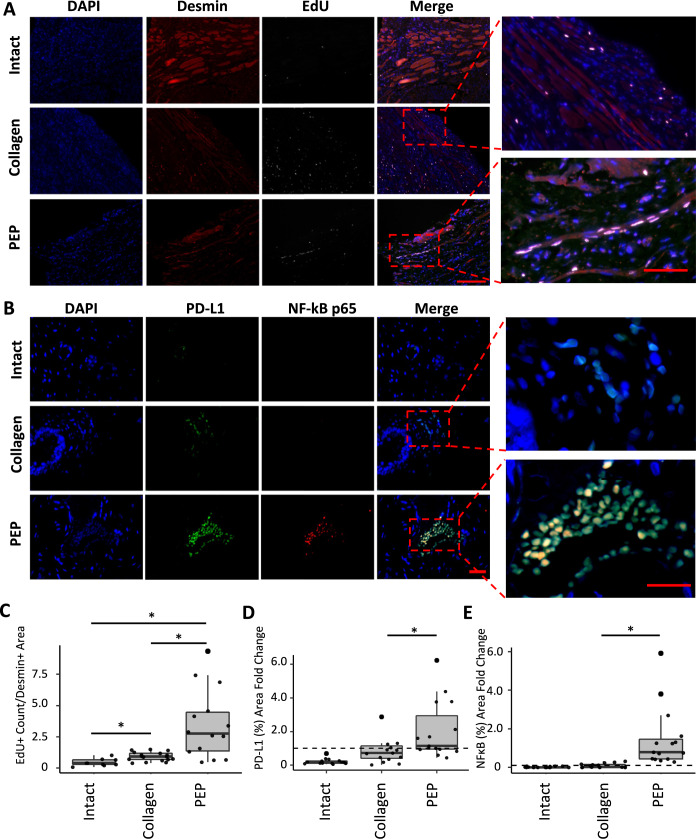
Immunohistochemical analysis of PEP-induced repair of urethral sphincter injury. Representative sections of urethral sphincter lesion repaired with collagen or collagen reconstituted with 1 × 10^12^ exosomes/mL PEP and intact urethra stained. **A** Nuclei were counterstained with DAPI (blue), skeletal muscle with Desmin (red), and EdU (white). Scale Bar = 100 µm. Magnified view of representative sections of intact urethra (Intact) and urethral sphincter lesion repaired with collagen (Collagen) or collagen reconstituted with 1 × 10^12^ exosomes/mL PEP (PEP). (*n* = 9, 18, 15 respectively). Scale bar = 30 µm. **C** ImageJ blinded quantification of de novo skeletal muscle proliferation estimated by calculating the number of EdU-positive cells per mm^2^ of Desmin-positive area. **B** Tissue was stained for PD-L1 (green), p65 NF-κB (red), and DAPI (blue). Merge overlay highlights PEP treated samples contained colocalization of PD-L1/NF-κB p65 staining of tissues. (*n* = 15, 15, 15) Scale = 20 µm. **D** ImageJ blinded quantification of PD-L1 expression by analysis of PD-L1-positive tissue area per total tissue area. **E** ImageJ blinded quantification of NF-κB p65 expression by analysis of NF-κB p65+ tissue area per total tissue area. *p < 0.05. Box-plot data represents the IQR (gray box) and whiskers represent 1.5 IQR. Student’s *t*-test was utilized.

Representative sections from both the injury site and intact segments of the urethra were stained for Desmin (a cytoskeletal intermediate filament found in sarcomeres of adult myoblasts)^48^, EdU, and DAPI to identify skeletal muscle, newly formed cells, and nuclei, respectively. While there was limited EdU / Desmin colocalization in the collagen-treated urethral lesion and intact urethral tissue (Fig. 6A), abundant colocalization of Desmin and EdU-positive cells was seen at the injury site injected with PEP (Fig. 6A). A magnified view of these areas revealed multinucleated cells staining positive for the above markers only in injury sites treated with PEP; suggesting de novo skeletal muscle cell regeneration with differentiation to multinucleated myotubes (Fig. 6A, zoomed inset). Quantification by a blinded observer documented significantly more EdU-positive cells per mm^2^ Desmin+ areas in injury sites treated with PEP compared to collagen control (3.46 ± 0.74 vs 0.92 ± 0.09; *p* < 0.001; Student’s *t*-test) and compared to the intact urethra (3.46 ± 0.74 vs 0.45 ± 0.10; *p* < 0.005; Student’s *t*-test) (Fig. 6C, Supplementary Table 7).

### Injury site NF-κB and PD-L1 expression and immune response elicited by PEP

**Fig. 7:**
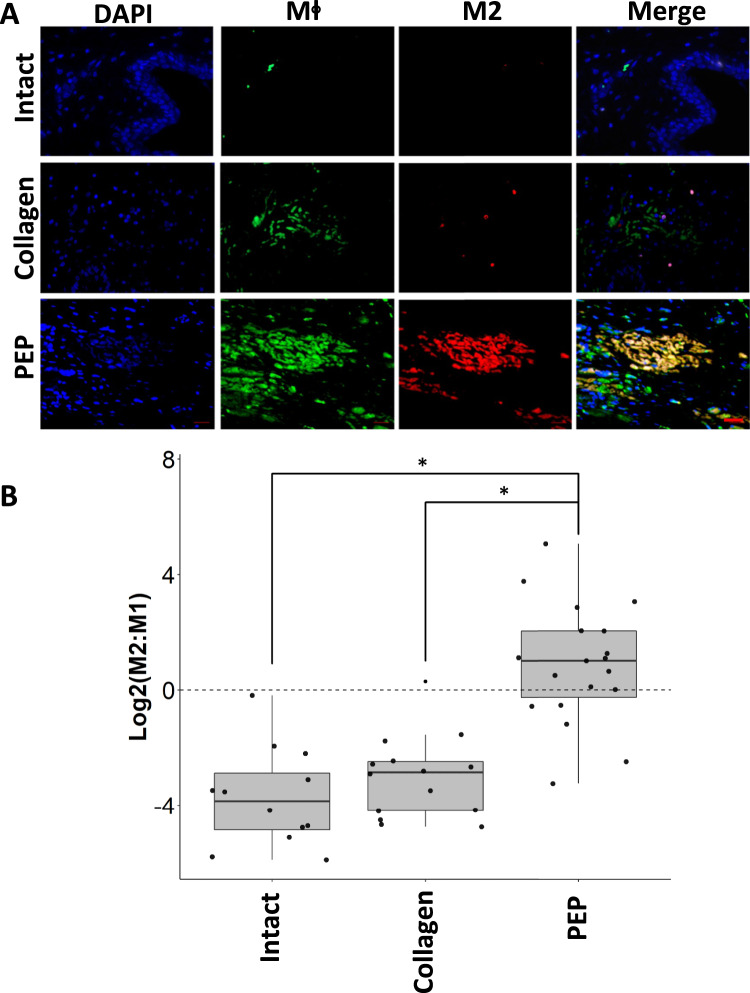
PEP Induces M2 macrophage polarization in a porcine SUI model. Representative sections of urethral sphincter lesion repaired with collagen, collagen reconstituted with 1 × 10^12^ exosomes/mL PEP or intact urethra. **A** Tissues were stained for general macrophage antigen (Mϕ; green), CD163 M2 specific macrophages (red), and DAPI (blue). Merge overlay highlights PEP samples contained colocalization of Mϕ + /CD163 + staining of tissues. Representative sections of intact urethra (Intact) and urethral sphincter lesion repaired with collagen (Collagen) or collagen reconstituted with 1 × 10^12^ exosomes/mL PEP (PEP). (*n* = 12, 15, 20, respectively) Scale = 20 µm. **B** ImageJ blinded quantification of Mϕ and M2 staining. M2:M1 ratio was determined from the comparison of image area of M2:(Mϕ–M2). A Haldane correction was utilized to account for division of 0 in M2:M1 ratio. The correction adds 0.5 to all values in the list to allow for the calculation to not yield an error. The graph represents the log base 2 ratio of M2:M1 macrophages. Dotted line at y = 0 shows the value at which the ratio would be equal. The log base 2 scale shows a M2:M1 > 1 at values Log2 > 0. *p < 0.05. Box-plot data represents the IQR (gray box) and whiskers represent 1.5 IQR. Student’s *t*-test was utilized.

With NF-κB and PD-L1 highly expressed in PEP, we hypothesized that sustained release would mediate local expression of these molecules and promote M2 macrophage polarization, an immune cell previously shown to be integral to skeletal muscle regeneration^49–51^. Accordingly, in sections of injured urethra treated with PEP, expression and colocalization of PD-L1 and NF-κB was detected (Fig. 6B) at significantly greater percentage when compared to collagen control for both PD-L1 (0.86 ± 0.19% PEP vs 0.42 ± 0.095% Collagen; *p* = 0.036) (Fig. 6B, D, Supplementary Table 8) and NF-κB (0.46 ± 0.13% PEP vs 0.032 ± 0.009% collagen; *p* = 0.002) (Fig. 6B, E, Supplementary Table 8). The intact urethra, lacking a triggering event, had limited macrophage staining for either population of immune cells (Figs. 6B and 7A). The macrophage response to collagen treatment appeared restricted to a less pronounced undifferentiated macrophage staining with a paucity of M2 macrophages (Figs. 6B and 7A). In contrast, an abundance of M2 predominant macrophages was found only in lesions treated with PEP (Fig. 7A). Quantification favored M2 polarization in PEP, but not collagen-treated lesions (M2:M1 ratio of 1.64 for PEP vs 0.21 for collagen control; *p* = 0.045) (Fig. 7B, Supplementary Table 9).

## Discussion

Extracellular vesicle involvement in skeletal muscle regeneration has previously been suggested; however, to our knowledge, the data presented herein are the first demonstration of a cell-independent exosome therapy implemented to restore muscle integrity. Our study evaluated the use of human platelet-derived exosomes (PEP) to stimulate myoblast growth in vitro and in two distinct in vivo models. A dose-dependent impact on myoblast proliferation, cell migration, and chemotaxis was observed. Resveratrol inhibition of PEP-mediated myoblast proliferation implicated a critical role for NF-κB p65; a molecule detected within PEP. In vivo, hydrogel-based sustain release of PEP led to significantly higher skeletal muscle repopulation in a VML latissimus dorsi model and improvement in urethral sphincter muscle function in a PEP treated porcine model of SUI. EdU and Desmin colocalization, sustained expression of NF-κB and PDL-1, in tandem with M2 macrophage polarization points to the in vivo activation of these established myoregenerative molecular cues within PEP treated tissues. Collectively, these findings provide proof of concept for the implementation of an exosome-based off-the-shelf acellular platform to restore skeletal muscle function.

Reservation following the 2008 and 2011 FDA public health warnings regarding the risk of mesh used for pelvic organ prolapse has led to a significant decline in the use of polypropylene mid-urethral sling, the most common procedure used worldwide to treat SUI. With the aging of the population, pelvic organ disease prevalence continues to be high^52^. The practice change in SUI has created a significant need to develop non-mesh-based alternatives^8,9,53,54^. SUI procedures focus on restoration of sub-urethral support, with failure of therapy thought to be likely related to suboptimal muscle coaptation rather than recurrent loss of support^54–57^. Presently, there are no clinically available solutions to permanently reverse incontinence and no effective interventions targeting the defective urethral sphincter. As such, recent efforts have targeted restoration of urethral sphincter function in order to improve continence^20,58–65^. Muscle precursor cells (MPC) applied to ablated urethral sphincter of older rats have been shown to reconstitute anatomic motor units and restore partial urethral function^20,66^. Phase 1 and 2 studies delivering MPC harvested from the quadriceps femoris reported no serious MPC-related adverse events and at higher cell concentrations provided signals of improvement in SUI symptoms^67,68^. Unfortunately, a phase 3 multicenter trial employing this intervention was halted due to a high placebo control response precluding efficacy comparisons^68^.

Although promising, widespread use of MPCs has significant limitations, including the morbidity of muscle biopsy, high manufacturing cost, dose-to-dose variability, and hurdles associated with cell transport and handling^69–72^. These limitations significantly inhibit the implementation of this approach not just within the United States but dramatically limit use in developing countries. The exosome technology described here provides a potential solution to these limitations as a scalable and room-temperature stable lyophilized product. In addition, by activating local myocyte progenitors, PEP eliminates the need for exogenous myoblast transplantation to drive desired regenerative events.

Satellite cells are mononucleated skeletal muscle stem cells that reside closely opposed to mature myofibers in a dormant state until they become activated by muscle injury. Upon activation, there is a cellular expansion to both replace the stem cell pool and repair damaged tissue. Cells destined for repair align along the injured fibers and differentiate into myotubes which fuse with the surrounding uninjured muscle to heal the injury^71^. Macrophages are pleotropic immune cells that play a critical role in muscle repair and regeneration^73,74^. Specifically, an orchestrated shift from early pro-inflammatory to wound healing and repair responses is necessary for muscle regeneration following injury. The former is mediated by M1 macrophages and is necessary for myofiber and wound debridement. The latter involves direct interactions between M2 macrophages and satellite cells at the site of myofiber injury^75^. With absent M2 polarization, there is aberrant skeletal muscle regeneration. Under local inflammatory conditions and during injury repair, myoblasts have been shown to express several B7-related (CD80 and CD86) co-stimulatory and inhibitory molecules, including PD-L1, the potent immune-modulator detected in large quantities in PEP^74,75^. In fact, an in vitro assay of myoblast regeneration under INF-ψ pro-inflammatory conditions showed marked upregulation of PD-L1^76^. Here, exogenous PD-L1, donated by PEP, was observed to drive a pro-regenerative transition from pro-inflammatory M1 to M2 macrophages in treated urethral sphincter sites.

Since the PEP utilized in this work was produced with adherence to current good manufacturing processes (CGMP) with defined purity, potency, and impurities^77–83^. Application of this technology within a translational large animal model provided both efficacy and toxicity data facilitating the first-in-man application of this platform. However, it should be highlighted that although large animal models of disease are the best surrogates for the human condition, they do not mimic actual degenerative disease. As such, although the work presented herein establishes PEP as a regenerative modality for skeletal muscle, its true impact on SUI necessitates in-human trial testing.

A limitation of our porcine model is that repair of an acute urethral sphincter muscle injury may not translate to clinical treatment of SUI; an ill-defined chronic condition that is believed to result from pelvic floor injury incurred at childbirth, striated muscle cell loss, denervation, and post-menopausal changes to urethral connective tissue and vasculature. An additional challenge to clinical translation is that the striated muscle changes along the longitudinal and ventral dorsal axis and with age, with older women having diminished muscle dorsally and in the mid to proximal ventral urethra. As mentioned above, satellite cells remain dormant until activated by injury. Although a focus the focus of this manuscript was on skeletal muscle and not peripheral nerve regeneration, previous work with PEP has shown its impact on the restoration of nerve function^77^. Hence, we have developed a device that will induce microinjuries along the length of the urethral sphincter that we believe will be critical for PEP potentiation of the endogenous skeletal muscle repair response. Encouraging preliminary continence data from trials injecting muscle precursor cells in the urethra provides proof of concept data that acute muscle regeneration may allow for reversal of chronic urethral sphincter defects associated with SUI in women^84^. Additionally, as collagen has been utilized as a treatment for SUI in women, collagen used in our study may have had a “bulking” effect rather than muscle regeneration. However, unlike bulking procedures in which the material is injected submucosally to obstruct the urethral lumen, our treatment involved injection of the biologic deep into the urethral wall with no cystoscopically observed “bulking” effect of the urethra. Moreover, treatment with collagen resulted in no observed changes in urethral pressure compared to day 7 post-injury values. Lastly, although a multiple-month long-term follow-up would be ideal, the porcine model utilized herein grows at such a pace that longer-term housing and evaluation were not feasible.

In summary, the highly stable, lyophilized PEP exosome product was here shown to drive myoblast proliferation and restore urethral sphincter muscle function following sustained release in vivo. As an off-the-shelf platform, this biologics-based therapy manufactured under CGMP controls for purity, potency, and sterility offers a new modality by which to restore skeletal muscle integrity. Cell-free products are more compatible with widespread deployment, however, because of a very short half-life, have typically not achieved significant benefit in clinical application. By generating a highly stable exosome platform, sustained released within a biopotentiated hydrogel, this manuscript provides evidence for a novel biologics-based modality to achieve mesh-free intervention in the treatment of stress urinary incontinence. The work presented herein underpins an upcoming clinical trial designed to establish the safety and efficacy signals of PEP therapy in women suffering from SUI.

## Methods

### Study design

The aim of this study was to assess the regenerative potential of the PEP. We were interested in developing a cell-free, off-the-shelf product that could restore urethral sphincter function and provide a non-mesh-based alternative surgical treatment for SUI. This platform has previously been tested in models of the tendon, nerve, vaginal mucosa, and ischemic wound healing^77–83^. Here, we characterized the in vitro Human Skeletal Muscle Myoblast proliferative, migratory, and chemotaxis response to PEP. Proliferation assays following pretreatment of myoblasts with increasing concentrations of resveratrol led to inhibition of myoblast growth and suggested that an exosome-dependent NF-κB p65 signaling pathway may be required for satellite cell activation and differentiation. Second, a volumetric muscle loss (VML) rat model was created to determine the in vivo efficacy of muscle regeneration and repair by PEP. Animals were randomized to saline, Tisseel, or Tisseel reconstituted with 1 × 10^12^ PEP exosomes/mL, and the repair response was characterized by Masson Trichrome and immunostaining against EdU and Desmin. Group sizes were dictated by IACUC protocols and were based on previous rat studies utilizing this model. As it was not clear if the newly regenerated muscle restored function and since urethral sphincter lesions causing SUI in humans are presumably less severe than the VML model, we developed a novel porcine SUI model by creating a targeted defect in the urethral sphincter. The created sphincter defect was sufficiently large to impair function and cause a significant decrease in urethral pressures. Animals underwent surgical repair of the urethral sphincter lesions with an injection of collagen or collagen with 1 × 10^12^ PEP exosomes/mL. We chose group sizes based on IACUC protocols and our laboratory’s previous experience with large animal experiments. The interventions were delivered cystoscopically or with a prototype injection device. Investigators were blinded when collecting and analyzing data but were not blinded to the treatment group during the injection of the active product. All animal studies were conducted under ethical approval by the Institutional Animal Care and Use Committee of Mayo Clinic, Rochester, MN

### Characterization and quantification of Purified Exosome Product

Purified Exosome Product (PEP; RION LLC, Rochester, MN) is purified from the conditioned medium of apheresis purified platelets^77–83^. Apheresis platelets from FDA-sanctioned blood banks were obtained, quantified at ~1.5 × 10^9^ platelets per ml from each donor, and maintained as a suspension culture in platelet additive medium (PAS III M, Grifols). The derived conditioned medium was harvested and pooled to achieve a total starting volume of 2 liters. Centrifugation and staged filtration from 100 µm down to 0.2 µm were performed to eliminate all cytological material and yield extracellular vesicles in the range of 50–200 nm, in CGMP adherent fashion. The presence of CD63, CD9, and Flotillin expression was documented in this starting material, in alignment with exosomal phenotypes with NanoSight NS3000 (Malveryn Panalytical). Under CGMP conditions, the filtered conditioned medium was aliquoted and next frozen at −80 °C and lyophilized (SP Scientific) in high vacuum conditions (~50 mTorr) in compatible stoppered vials (Corning) to yield a cake containing ~5 × 10^12^ vesicles per vial^80–82^, no excipients were added in this process. Different exosome concentrations were created by suspending the cake in different volumes of saline or in the described suspension media. Nanoparticle tracking analysis was conducted on the NanoSight NS3000 to characterize the particle size distribution and quantify exosome concentration. Electron microscopy TEM) was conducted at a Mayo Clinic Microscopy and Cell Analysis Core Facility on a JEM-1400 Series 120 kV microscope (JEOL) with an acceleration voltage of 80 kV and an indicated magnification of ×60k. TEM was conducted with PEP in solution, Tisseel or collagen, and Tisseel or collagen reconstituted with 1 × 10^12^ PEP exosomes/mL.

### PEP biodistribution following intramuscular injection

PEP vesicles were labeled with near-far red DiR (Thermo) according to the manufacturer’s protocol. Fifty micromolar DiR was incubated with 5 × 10^12^ exosomes at 37 °C for 10 min. After, the DiR labeled exosomes were washed twice with lactated ringers by use of a 30 kDa Amicon column filter (Sigma) at 4000 × *g* for 15 min. Three mice were injected intramuscularly into the right quadriceps with 100 µL of DiR labeled PEP (5 × 10^12^ exosomes/mL). Sequential assessment of PEP biodistribution was assessed by the Xenogen IVIS Spectrum (PerkinElmer). In vivo distribution was assessed over 53 h post-injection, followed by imaging of excised and isolated organs. All images were collected with an exposure time of 10 s, an excitation filter of 745 nm, and an emission filter of 800 nm.

### Antibodies utilized

For western blotting immunodetection the following antibodies were used: NF-κB (1:1000, Santa Crus, SC-8008), PD-L1 (1:1000, Cell Signaling, #13684 S), CD63 (1:1000, R&D Systems, MAB50482), CD9 (1:1000, Cell Signaling Technology, 13403 S), Flotillin-1 (1:1000, Abcam, ab133497), and Actin (1:1000, Licor, 926–42212).

For immunocytochemistry and immunohistochemistry applications the following antibodies were used: Desmin (1:250, Abcam, ab32362), NF-κB (1:200, Santa Crus, SC-8008), PD-L1 (1:200, Cell Signaling, #13684 S), Mφ (1:100, LifeSpan BioScience, LS-B9966), CD163 (1:200, Abcam, ab87099), Pax7 (1:100, ThermoFisher, PA1-117), MyoD (5.8 A) (1:200, ThermoFisher, MA1-41017), and Heavy Chain Cardiac Myosin (1:100, Abcam, ab50967). A complete table of antibodies can be found in Supplemental Table 10.

### Western blotting technique and quantification

In a digital fluorescence western blotting (Li-Cor Biosciences) method, equal amounts of protein were loaded into each lane and transferred to a nitrocellulose membrane. The membranes were blocked in Li-Cor TBS Blocking Buffer and incubated with primary antibodies against CD9, CD63, Flotillin-1, Actin, NF-κB p65, and PD-L1. Secondary antibodies at 680 nm and 800 nm were used to fluorescently image the membranes using the Li-Cor Oddyssey CLx. Digital fluorescent intensity was determined by Image Studio Software, and loading was standardized by Li-Cor total protein stain for whole lane protein digital fluorescence and analyzed by Empiria Studio Software. All raw unedited blots are included in Supplementary Fig. 9.

### Atomic-force microscope (AFM) and single-particle interferometric reflectance imaging sensing (SP-IRIS) analysis

AFM was performed as previously described^85^. Briefly, contact-mode AFM with silicon nitride NP-S tips (spring constant, 0.58 Newton/meter) was performed on a Nanoscope III controller (Digital Instruments). Exosomes either purified through traditional ultracentrifugation or through the methodology described above (PEP) were fixed in situ, rinsed with ultrapure water (18 MΩ-cm), and dried. Images were obtained using linear scanning frequencies (5–15 Hz) to generate 512 × 512-pixel AFM images. Three-dimensional topographical images were generated and quantified using Nanoscope software.

Two lots, in triplicate, of lyophilized PEP were reconstituted in 1 ml Incubation Solution I (NanoView Biosciences) and further diluted 1000-fold in the same solution prior to incubating 50 μl on-chip, for 16 h according to the manufacturer’s protocol for the ExoView human tetraspanin plasma kit (NanoView Biosciences; EV-TETRA-P). ExoView tetraspanin plasma chips are spotted with capture antibodies against CD41a and the mouse IgG1,κ isotype control in triplicate. The chips were then washed in an automated chip washer and incubated with conjugated antibodies for fluorescent labeling of the captured PEP (CF488a anti-CD9, NanoView Biosciences) for 1 h After labeling, the chips were washed and dried in the automated chip washer and placed in the reader for analysis. All data were gathered using an ExoView R100 reader equipped with ExoView Scanner 3.0 software and analyzed using ExoView Analyzer 3.0. All data reported was obtained and analyzed in a blinded fashion adhering to CGMP quality-controlled standards.

### HUVEC internalization of PKH26 labeled PEP

PEP vesicles were labeled with PKH26 (Sigma) according to the manufacturer’s protocol. 5 × 10^12^ vesicles were resuspended in Diluent C, mixed with 4 µL of PKH26, and incubated at room temperature for 5 min. Labeling was quenched by the addition of 2 mL 10% BSA (Sigma) and 8.5 mL serum-free medium (Lonza). Labeled PEP was then concentrated by centrifugation at 3000 × *g* with an Amicon 10 kDa filter column (Sigma). HUVECs (Lonza) were cultured with EBM^TM^-2 Endothelial Cell Growth Basal Medium with EGM^TM^-2 Endothelial Cell Growth Medium-2 Bullet Kit^TM^ (Lonza). To assess the internalization of PEP, HUVECs were cultured in the presence of PKH26 labeled vesicles for 18 h. Cells were imaged using an LSM 780 confocal microscope (Carl Zeiss).

### Characterization of the effect of PEP on human skeletal muscle myoblasts

Human Skeletal Muscle Myoblasts (HSMM, Lonza, CC-2580) growth, migratory and chemotaxis properties were assessed with cell culture with basal media (SkBM-2 Skeletal Muscle Cell Growth Basal Media, Lonza, CC-3246), basal media supplemented with 10% FBS (SkGM-2 SingleQuots Supplements and Growth Factors, Lonza, CC-3244) or basal media with increasing concentrations of PEP ranging from 1.25 × 10^11^ exosomes/mL to 5 × 10^11^ exosomes/mL was assessed using IncuCyte Live-Cell Analysis (Essen Bioscience). Data was generated from IncuCyte Live-Cell analysis by acquiring phase images (×10 lens; 4 images/well in 96-well plate with 12 wells/condition).

Chemotactic properties of PEP were assessed using Chemotaxis module on IncuCyte. HSMM in media were plated on the top chamber, and chemotaxis was assessed by placing basal media, media supplemented with 10% FBS, or media with increasing concentrations of PEP ranging from 1.25 × 10^11^ exosomes/mL to 5 × 10^11^ exosomes/mL in the bottom chamber. Images were collected from both the top and the bottom chambers. The total phase area in the top well was normalized to the initial top value with a decrease in cell area in the top chamber, indicating enhanced cell migration through the pore and improved chemotaxis. The graph was plotted using IncuCyte Live-Cell chemotaxis module by acquiring wide-angle phase images (×10 lens; 1 image/well in 96-well plate with 12 wells/condition).

HSMM migration was assessed using a scratch wound assay. The graph is plotted at different time points with basal media, media supplemented with 10% FBS, and media with increasing concentrations of PEP ranging from 1.25 × 10^11^ exosomes/mL to 5 × 10^11^ exosomes/mL using IncuCyte. Data is generated from IncuCyte Live-Cell analysis by acquiring phase images (×10 lens; 1 image/well in 96-well plate with 12 wells/condition).

### Resveratrol inhibition of human skeletal muscle myoblast proliferation

The mechanism of PEP-induced HSMM proliferation was assessed using resveratrol (Invivogen); an NF-κB inhibitor, in a culture grown with 2.5 × 10^11^ exosomes/mL PEP. This concentration was chosen both because of its optimal growth and because of the empirically determined timed release PEP concentration from Tisseel reconstituted with 1 × 10^12^ exosomes/mL PEP (Table S3). HSMM were plated on a 96 well at 10k cells/well overnight. Resveratrol was incubated at concentrations ranging from 50 to 500 µM for an hour before adding 2.5 × 10^11^ exosomes/mL PEP to each well. Data are generated from IncuCyte Live-Cell Proliferation Analysis by acquiring phase images (×10 lens; 5 image/well in 96-well plate with 3 wells/condition). Li-Cor western blotting for expression of NF-κB p65 at 72 h post-inhibition was quantified using Image Studio Software and normalized using a total protein stain.

### Exosome release assay

For the Tisseel release assay, a Tisseel kit (Baxter, #1504517) was prepared by adding 1 mL of Fibrinolysis Inhibitor to Sealer Protein Concentrate and adding 1 mL of CaCl_2_ Solution to Thrombin 500. Both solutions were incubated for 20 min at 37 °C. One vial of PEP (Lot# 19004-B1) was resuspended with 400 uL heparin and 1.25 mL deionized water. The entire PEP solution was added to the Fibrinolysis Inhibitor/Sealer Protein Concentrate Solution. This solution was combined in each well of a 12-well plate in a 2:1 ratio with the CaCl_2_/Thrombin 500 solution for a total volume of 990 ul per well.

For the Collagen release assay, one vial of PEP (Lot# 19004-B1) was reconstituted in 2.5 mL of sterile water and filtered through a 0.22 um filter. Five hundred microliters PEP was combined with 500 uL of 6 mg/mL collagen (Collagen Solutions FS22004). NaOH was added to a final concentration of 0.02 M. The entire 1 mL solution was added to a single well of a 12-well plate and incubated at 37 °C until solid. The final concentration of PEP for both assays in each well was 1 × 10^12^ exosomes/mL. One mL of serum-free Dulbecco’s Modified Eagle Medium 1× (DMEM, Corning, 10-013CV) was added to each well. All media was collected daily and replaced with another 1 mL of serum-free DMEM for the time-course of the experiment.

### Repair of a Rat Latissimus Dorsi volumetric muscle loss model

After an acclimation period of 48–72 h, 27 2–4-month-old male Lewis rats (Envigo) were housed and handled according to the Mayo Clinic Institutional Animal Care and Use Committee (IACUC) regulations (Mayo Clinic, Rochester, MN). Animals were clipped and anesthetized via isoflurane inhalation prior to surgery and maintained by intravenous fluids and isoflurane for the duration of the procedure. After the skin was prepped with betadine, a 3 cm longitudinal incision was made above the scapula, the skin mobilized with Metzembaum scissors, and the latissimus dorsi (LD) muscle was exposed. An 8 mm punch biopsy was used to create the LD volumetric muscle loss, taking care to not exceed ~2 mm depth to avoid entering the chest cavity. The defect was filled with saline, Tisseel (Baxter), or Tisseel reconstituted with 1 × 10^12^ exosomes/mL PEP. The skin was closed with an interrupted 2.0 Vicryl suture. 5-ethynyl-2CE-deoxyuridine (EdU) was administered intraperitoneally (IP) at a dosage of 50 mg/kg once per week as a means of tracking cell proliferation. Animals were sacrificed at 6 weeks with Fatal-Plus intravenously (100 mg/kg).

### Directed urethral sphincter defect porcine model to assess the efficacy of PEP-induced sphincter muscle regeneration

After an acclimation period of 48–72 h, 10 female domestic Yorkshire-Crossed pigs, weighing 70–80 Kg, underwent two survival surgeries and one terminal surgery. All animals underwent the outlined procedures, although their injected intervention differed as described below.

Anesthesia for survival surgery #1 was induced with Telazol (5 mg/kg), Xylazine (AnaSed; 1–2 mg/kg), and maintained with Isofluorane-vaporizer (1.5–3%). After obtaining general anesthesia, the pig was prepared and draped in a sterile fashion. Ceftiofur (5 mg/kg) was given intramuscularly for antibiotic prophylaxis. Cystoscopy was performed, the urethra identified, and a whistle tip stent placed. Using cystoscopic guidance, the Medspira catheter was placed in the urethra. The stent was removed, the bladder balloon inflated to 30 cc, and pressures were obtained starting in the vagina and extending to the bladder neck in 1 cm increments (Supplementary Fig. 7). Pressures were collected for 10 s at each point, and 3 pressures along 10 s recordings were averaged (Supplementary Fig. 7). The pressure catheter balloon was deflated, the catheter removed, and the stent used to reidentify the urethra. Using Collin’s knife, a 2–3 cm long, full thickness trans-mural defect involving the urethra wall and underlying sphincter muscle was created ~1 cm cephalad of the meatus (cut setting at 70 watts). Light hemostasis was obtained with Collin’s knife (coagulation setting at 30 watts). A Foley catheter was placed for 48–72 h to prevent urinary retention. The position was confirmed cystoscopically, and the catheter was secured with a 2.0 Prolene suture. The animals were allowed to heal for a ~7 days and returned to the operating room for the intervention procedure. Buprenorphine (0.03 mg/kg) was given every 6–8 h for analgesia.

Anesthesia for survival surgery #2 was obtained as described above. Cystoscopy was used to identify the urethra, and the Medspira catheter was used to measure pressures as previously described (Supplementary Fig. 7). After pressure readings were collected, cystoscopy was used to inject the length of the previously created defect with collagen and 1 × 10^12^ exosomes/mL PEP (*n* = 4) or collagen alone (*n* = 2). A 1 × 10^12^ exosomes/mL PEP gel was created by reconstituting PEP (RION LLC, Rochester, MN) in 1 mL of sterile water (Hospira Inc., Lake Forest, IL) and 4 mL of clinical-grade type I bovine collagen (5 mg/mL; Collagen Solutions, Glasgow, UK). Collagen was made by adding 1 ml of sterile water to 4 ml of clinical-grade type I bovine collagen (5 mg/mL; Collagen Solutions, Glasgow, UK). A total of 5 ml was injected in ~0.5 ml aliquots along the length of the lesion using Injetak 21-gauge needle. A prototype PEP delivery device was also used to deliver 1 × 10^12^ exosomes/mL PEP + Collagen (*n* = 2) and collagen alone (*n* = 2). This device allows for the deployment of 2 needles at the 4 and 8 o’clock positions at specific points along the length of the urethra and is being developed to avoid the need for cystoscopy or general anesthesia. The device, specially modified for the porcine anatomy, was placed in the urethra, the needles deployed, 1 cc injected, and the needles retracted. This process was repeated five times, starting ~1 cm from the urethra meatus and extending the cephalad. The position of the device in the urethra was confirmed cystoscopically.

To aid in tracking cell populations, 2’-Deoxy-5-ethynyluridine (EdU) was given as an oral supplement (5 mg/Kg) twice weekly. After 6–7 weeks of healing, terminal surgery was performed. After anesthesia was obtained, the urethra was identified, urethral pressures were collected, and animals were euthanized using intravenous Pentobarbital (250 ml of 390 mg/ml). The perineum was removed en-block, and the urethra and bladder were placed in formalin. Organs were weighed, and representative samples were collected. Blood draws were collected, and chemistry was obtained.

### Histological procedure and staining quantification

Formalin-fixed samples embedded in paraffin were processed on a microtome into 10 µm sections. Tissues were stained with Hematoxylin and Eosin Stain (H&E) and Masson’s Trichrome Stain and were assessed using the Axio Scan.Z1 Slide Scanner (Zeiss). Additional sections were de-paraffinized in sequential xylene washes and rehydrated in decreasing amounts of ethanol baths, finally being washed in water. Antigen retrieval was conducted by submerging sections into a sodium citrate buffer (10 mM sodium citrate, 0.05% Tween 20, pH 6.0) and boiling for 10 min in a pressure cooker. Sections were then permeabilized with blocking buffer (PBS + 5% normal donkey serum, 5% BSA, 0.2% Triton-X) for an hour at room temperature. Primary antibodies against Desmin, PD-L1, NF-κB p65, MФ, and M2 macrophages were diluted in a blocking buffer overnight at 4 °C (Table S10). Then, AlexaFluor® (ThermoFisher Scientific) secondary antibodies were diluted 1:500 in blocking buffer and incubated for one hour at room temperature. EdU was labeled according to the manufacturer’s instructions using the Click-iT Plus EdU AF647 Imaging Kit (ThermoFisher Scientific, C10640). Following washes, ProLong Gold Antifade Mountant with DAPI® (ThermoFisher Scientific, P36935) was added to sections, a cover glass applied and imaged on a Axio Observer inverted fluorescent microscope (Zeiss) with variable fluorescence objectives. Gross images were taken with an iPhone camera.

Localization of the injury border zone of the injury was confirmed by Masson’s Trichrome stain in order to correctly image the immunohistochemistry. Quantification of immunohistochemistry was assessed using ImageJ (Version 1.52a) macro scripts in a blinded manner. Tissues stained positive for Desmin, PD-L1, and NF-κB p65 were quantified per percent tissue area of each respective image. Characterization of M2:M1 macrophage staining was conducted by quantifying MФ and M2 area percentage per tissue area of each image. M1 quantification was defined by MФ-positive tissue minus M2-positive macrophage-stained tissue. Additionally, EdU-positive count determined by object count per Desmin-positive stained tissue area was quantified for determination of proliferative muscle.

### External urethral sphincter pressure collection

Urethral pressures were assessed using a portable mCompass Anorectal Manometry system (Medspira, Minnesota, USA) with a 5-channel modified pressure catheter using the squeeze mode of the biofeedback 1.01 software (Fig. 5D and Supplementary Fig. 8). Pressure recordings were performed in triplicate and were assessed at four time points: pre-injury, post-injury, prior to injection, and prior to sacrifice (Day 0, 0, 7, and 42, respectively; Supplementary Tables 4–6). Pressure readings were obtained along the length of the genitourinary tract, starting in the vagina and extending to the bladder neck in 1 cm increments (Supplementary Fig. 7). At each point, pressures were collected for 10 s, and three pressures along a given 10 s recordings were averaged (Supplementary Fig. 7). Entry into the urethra was determined by a change in resistance with the advancement of the pressure sensor and an associated rise in urethral pressures. Urethral entry and pressure measurement location were confirmed cystoscopically.

### Statistical analysis

Data were reported as mean ± standard error of the mean and interquartile range and statistical significance assessed with two-tail student’s *t*-test or assessed by single variable ANOVA with post-hoc Tukey Honestly Significant Difference Test comparing all experimental groups. Significance was set to the alpha of 0.05.

### Reporting summary

Further information on research design is available in the Nature Research Reporting Summary linked to this article.

## Supplementary information

SUPPLEMENTAL FIGURES 1-9, SUPPLEMENTAL FIGURE LEGEND

Supplemental Movie 1

REPORTING SUMMARY

## Data Availability

All experimental data details, beyond what is in the Methods section that is deemed necessary to reproduce findings from this manuscript will be made available at request to interested investigators by contacting the corresponding author(s) by email at the addresses provided.

## Code availability

No code was utilized in this manuscript.
