# Author Correction: Exosome biopotentiated hydrogel restores damaged skeletal muscle in a porcine model of stress urinary incontinence

**DOI:** 10.1038/s41536-022-00260-5

**Published:** 2022-10-31

**Authors:** Tyler J. Rolland, Timothy E. Peterson, Raman Deep Singh, Skylar A. Rizzo, Soulmaz Boroumand, Ao Shi, Tyra A. Witt, Mary Nagel, Cassandra K. Kisby, Sungjo Park, Lois A. Rowe, Christopher R. Paradise, Laura R. E. Becher, Brooke D. Paradise, Paul G. Stalboerger, Emanuel C. Trabuco, Atta Behfar

**Affiliations:** 1 Van Cleve Cardiac Regenerative Medicine Program, Mayo Clinic Center for Regenerative Medicine, Rochester, MN USA; 2 Mayo Clinic Department of Cardiovascular Medicine, Rochester, MN USA; 3 Mayo Clinic Medical Sciences Training Program, Rochester, MN USA; 4 Mayo Clinic Department of Molecular Pharmacology and Experimental Therapeutics, Rochester, MN USA; 5 Mayo Clinic Division of Urogynecology, Rochester, MN USA; 6 Marriott Heart Disease Research Program, Mayo Clinic, Rochester, MN USA; 7 Rion LLC, Rochester, MN US

**Keywords:** Regenerative medicine, Translational research

Correction to: *npj Regenerative Medicine* 10.1038/s41536-022-00240-9, published online 29 September 2022

In this article the author name Raman Deep Singh was mistakenly provided as Ramandeep Takhter. This has now been amended.

