# Supplementary material for: Exosome biopotentiated hydrogel restores damaged skeletal muscle in a porcine model of stress urinary incontinence
Source: NPJ Regen Med. 2022 Sep 29;7:58. doi: 10.1038/s41536-022-00240-9 (PMC9523025; doi:10.1038/s41536-022-00240-9)
Supplement: Supplementary file 1 — SUPPLEMENTAL FIGURES 1-9, SUPPLEMENTAL FIGURE LEGEND [file 41536_2022_240_MOESM1_ESM.pptx]

## Slide 1
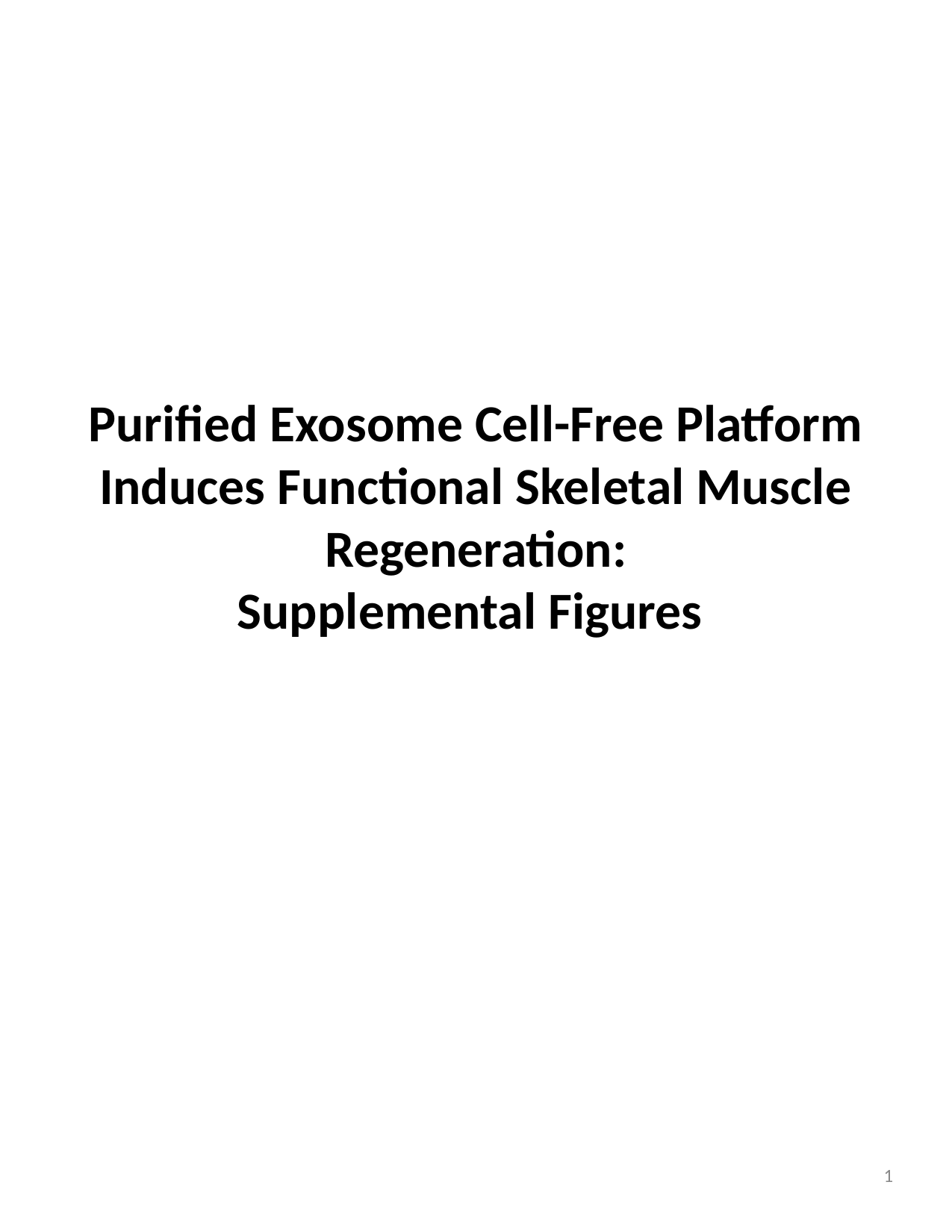

# Purified Exosome Cell-Free Platform Induces Functional Skeletal Muscle Regeneration: Supplemental Figures
1

## Slide 2
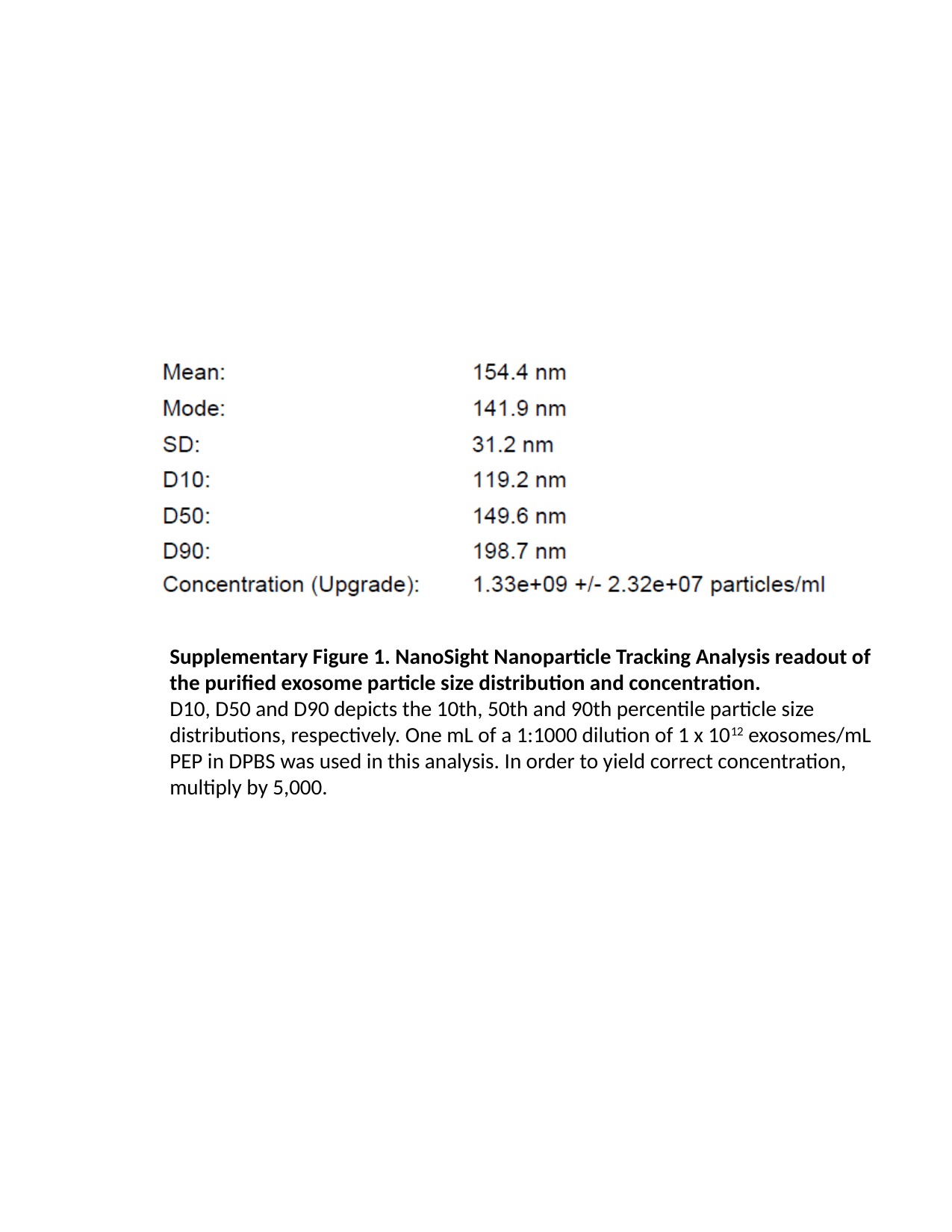

Supplementary Figure 1. NanoSight Nanoparticle Tracking Analysis readout of the purified exosome particle size distribution and concentration.
D10, D50 and D90 depicts the 10th, 50th and 90th percentile particle size distributions, respectively. One mL of a 1:1000 dilution of 1 x 10^12^ exosomes/mL PEP in DPBS was used in this analysis. In order to yield correct concentration, multiply by 5,000.

## Slide 3
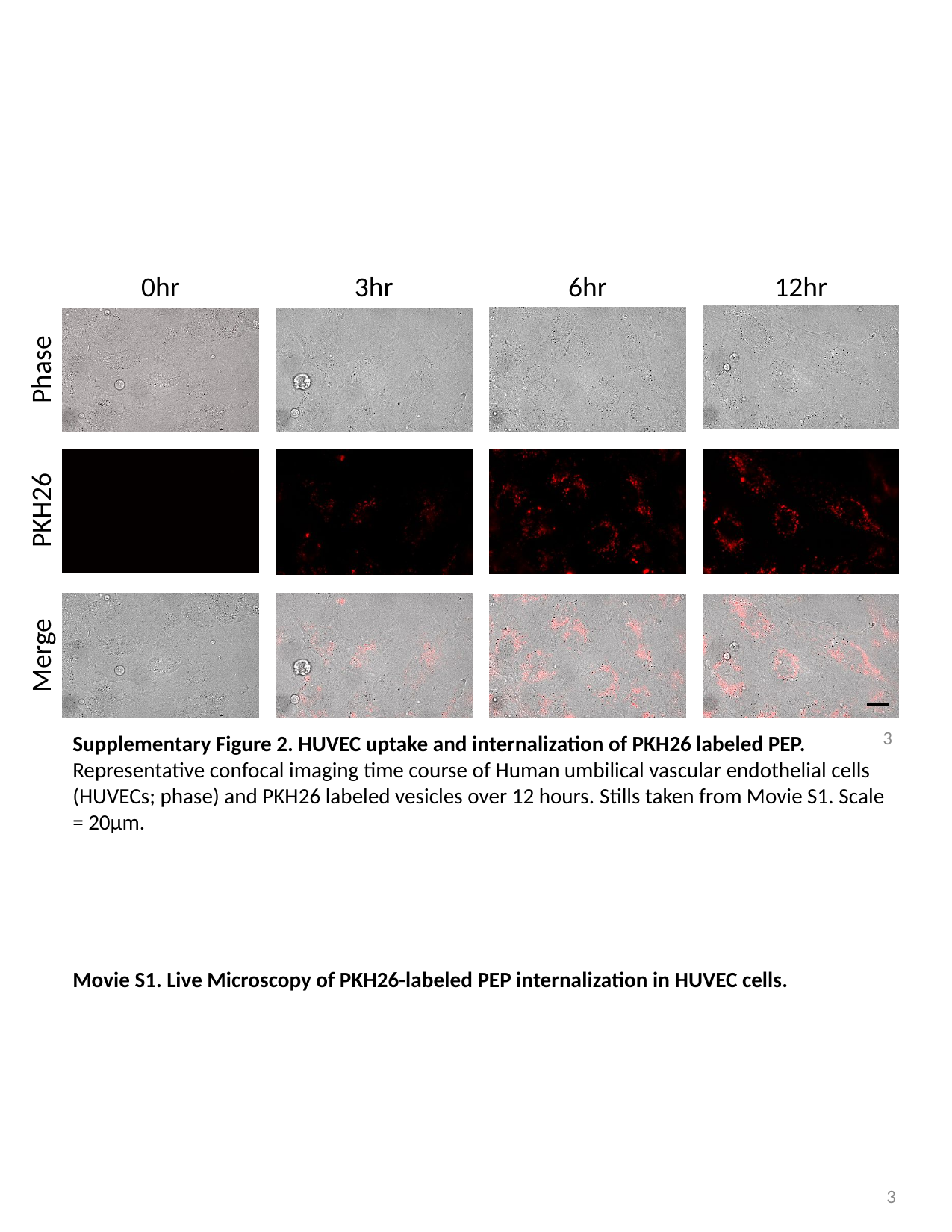

0hr
3hr
6hr
12hr
Phase
PKH26
Merge
3
Supplementary Figure 2. HUVEC uptake and internalization of PKH26 labeled PEP. Representative confocal imaging time course of Human umbilical vascular endothelial cells (HUVECs; phase) and PKH26 labeled vesicles over 12 hours. Stills taken from Movie S1. Scale = 20µm.
Movie S1. Live Microscopy of PKH26-labeled PEP internalization in HUVEC cells.
3

## Slide 4
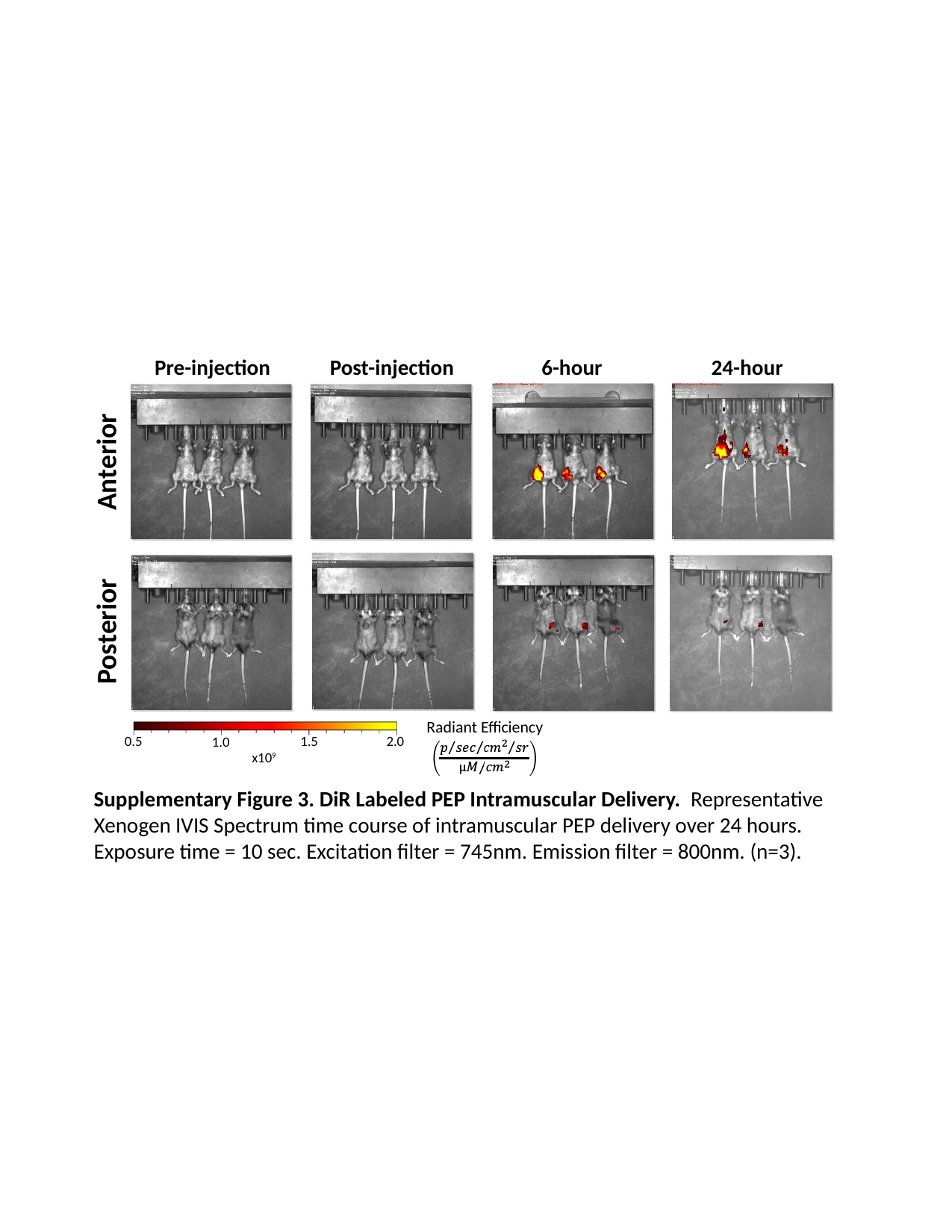

Pre-injection
Post-injection
6-hour
24-hour
Anterior
Posterior
Radiant Efficiency
0.5
1.5
2.0
1.0
x109
Supplementary Figure 3. DiR Labeled PEP Intramuscular Delivery. Representative Xenogen IVIS Spectrum time course of intramuscular PEP delivery over 24 hours. Exposure time = 10 sec. Excitation filter = 745nm. Emission filter = 800nm. (n=3).

## Slide 5
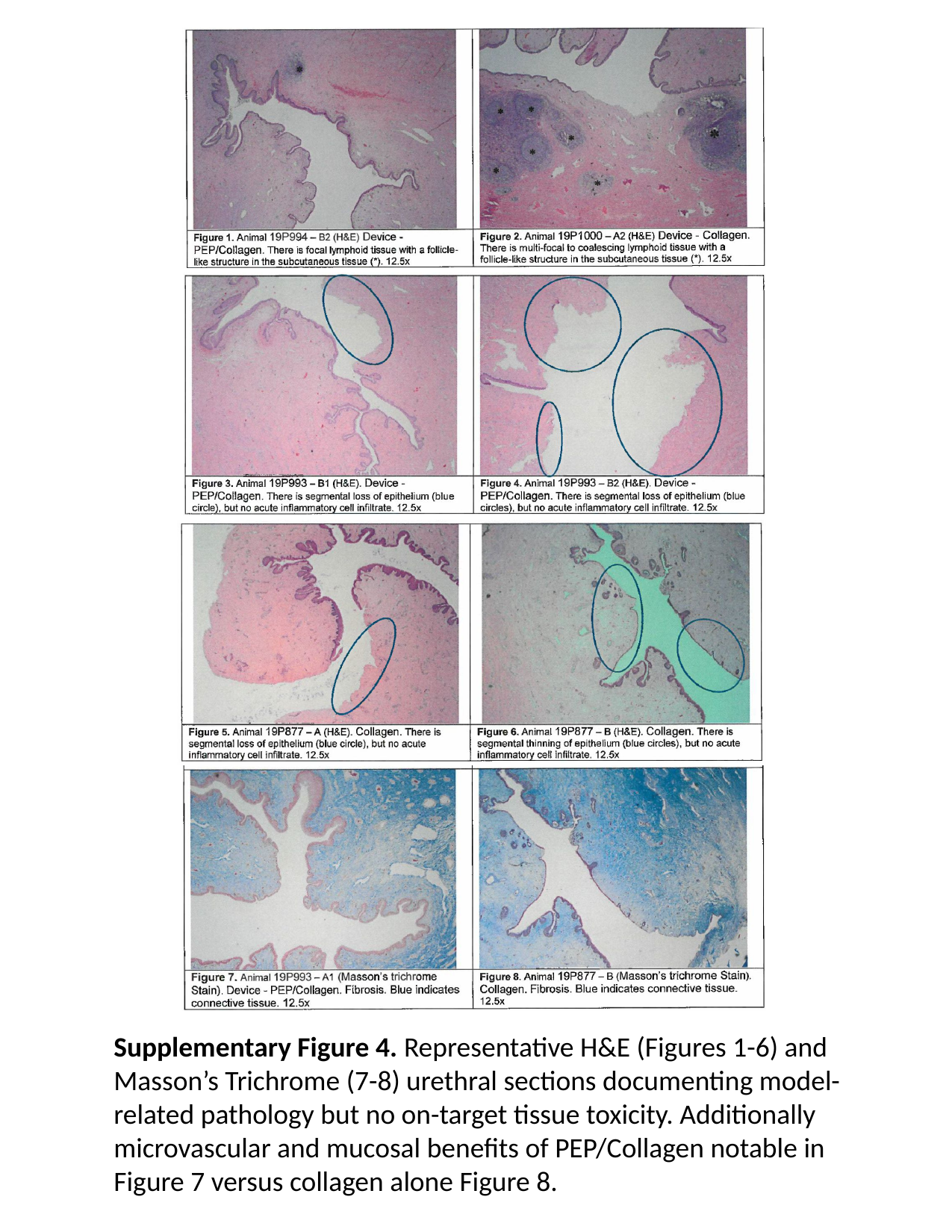

Supplementary Figure 4. Representative H&E (Figures 1-6) and Masson’s Trichrome (7-8) urethral sections documenting model-related pathology but no on-target tissue toxicity. Additionally microvascular and mucosal benefits of PEP/Collagen notable in Figure 7 versus collagen alone Figure 8.

## Slide 6
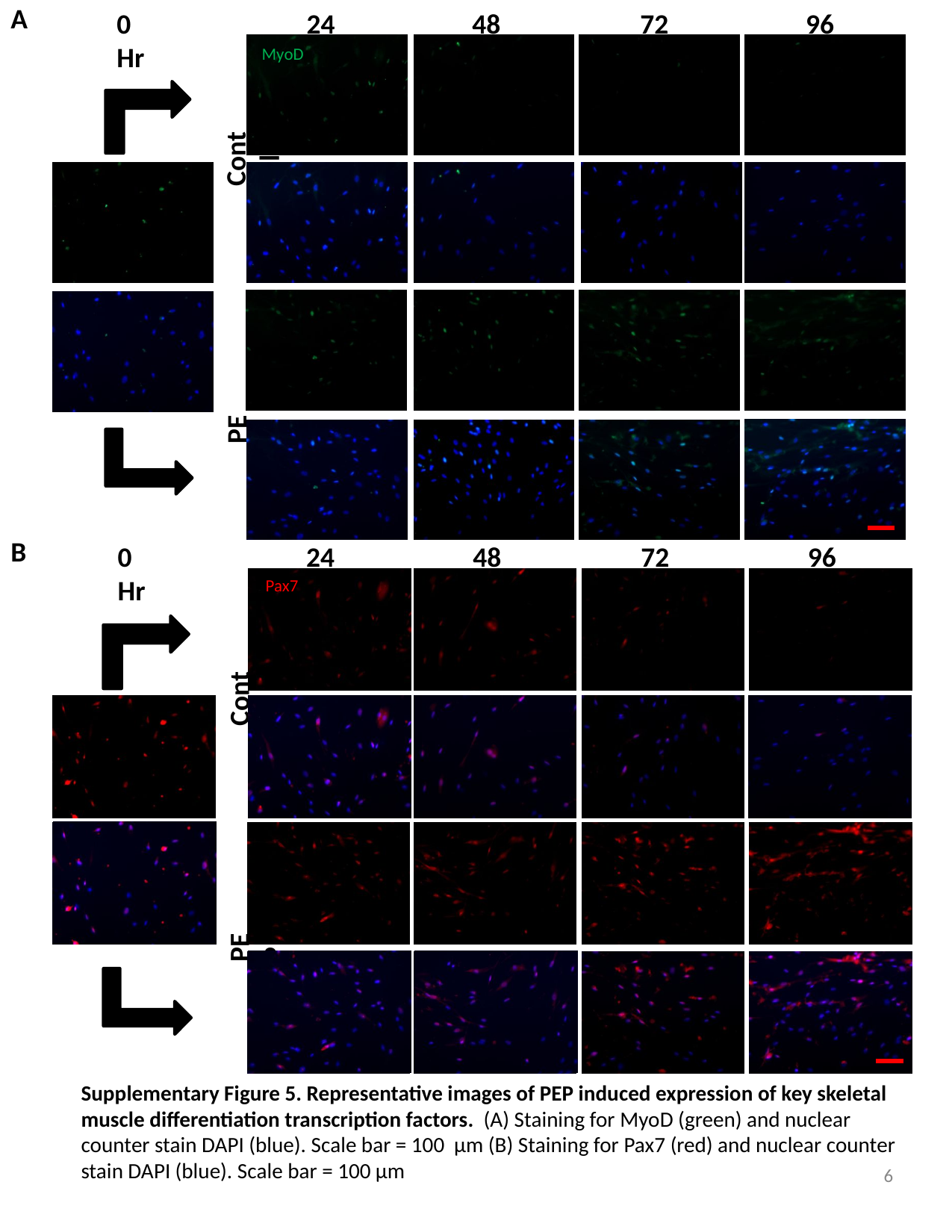

0 Hr
24 Hr
48 Hr
72 Hr
96 Hr
Control
PEP
A
MyoD
B
0 Hr
24 Hr
96 Hr
48 Hr
72 Hr
Control
PEP
Pax7
Supplementary Figure 5. Representative images of PEP induced expression of key skeletal muscle differentiation transcription factors. (A) Staining for MyoD (green) and nuclear counter stain DAPI (blue). Scale bar = 100 µm (B) Staining for Pax7 (red) and nuclear counter stain DAPI (blue). Scale bar = 100 µm
6

## Slide 7
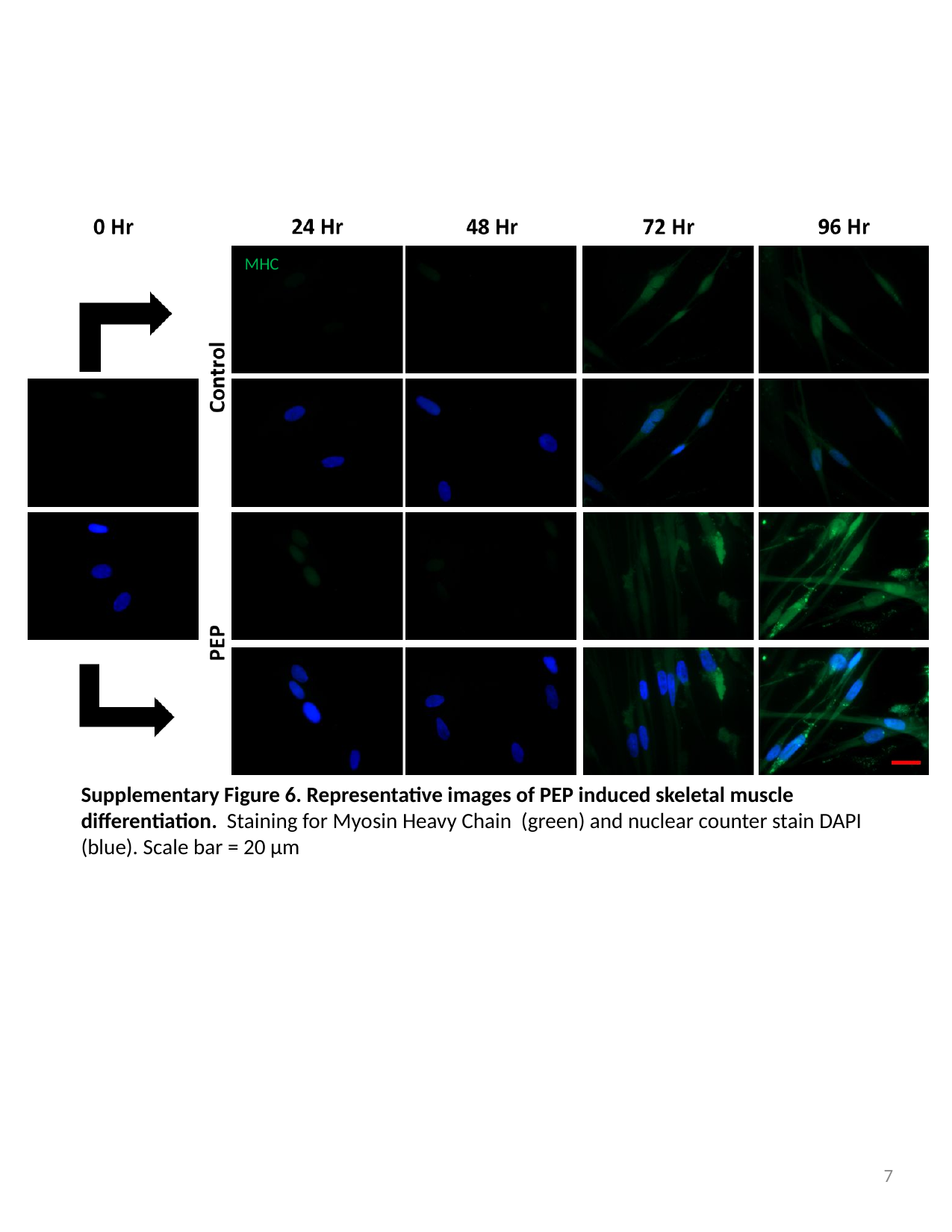

MHC
Supplementary Figure 6. Representative images of PEP induced skeletal muscle differentiation. Staining for Myosin Heavy Chain (green) and nuclear counter stain DAPI (blue). Scale bar = 20 µm
7

## Slide 8
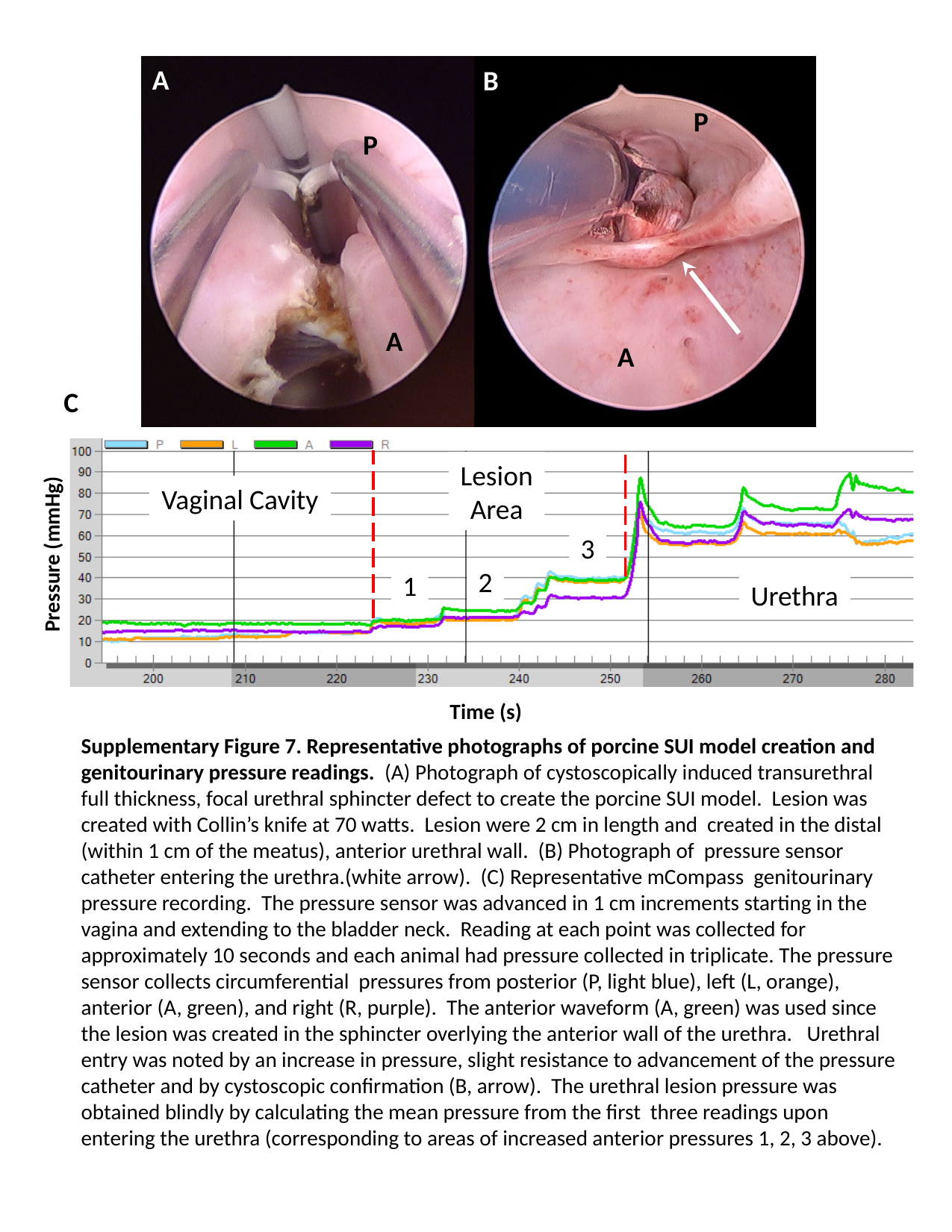

A
B
P
P
A
A
C
Lesion
Area
Vaginal Cavity
3
Pressure (mmHg)
2
1
Urethra
Time (s)
Supplementary Figure 7. Representative photographs of porcine SUI model creation and genitourinary pressure readings. (A) Photograph of cystoscopically induced transurethral full thickness, focal urethral sphincter defect to create the porcine SUI model. Lesion was created with Collin’s knife at 70 watts. Lesion were 2 cm in length and created in the distal (within 1 cm of the meatus), anterior urethral wall. (B) Photograph of pressure sensor catheter entering the urethra.(white arrow). (C) Representative mCompass genitourinary pressure recording. The pressure sensor was advanced in 1 cm increments starting in the vagina and extending to the bladder neck. Reading at each point was collected for approximately 10 seconds and each animal had pressure collected in triplicate. The pressure sensor collects circumferential pressures from posterior (P, light blue), left (L, orange), anterior (A, green), and right (R, purple). The anterior waveform (A, green) was used since the lesion was created in the sphincter overlying the anterior wall of the urethra. Urethral entry was noted by an increase in pressure, slight resistance to advancement of the pressure catheter and by cystoscopic confirmation (B, arrow). The urethral lesion pressure was obtained blindly by calculating the mean pressure from the first three readings upon entering the urethra (corresponding to areas of increased anterior pressures 1, 2, 3 above).

## Slide 9
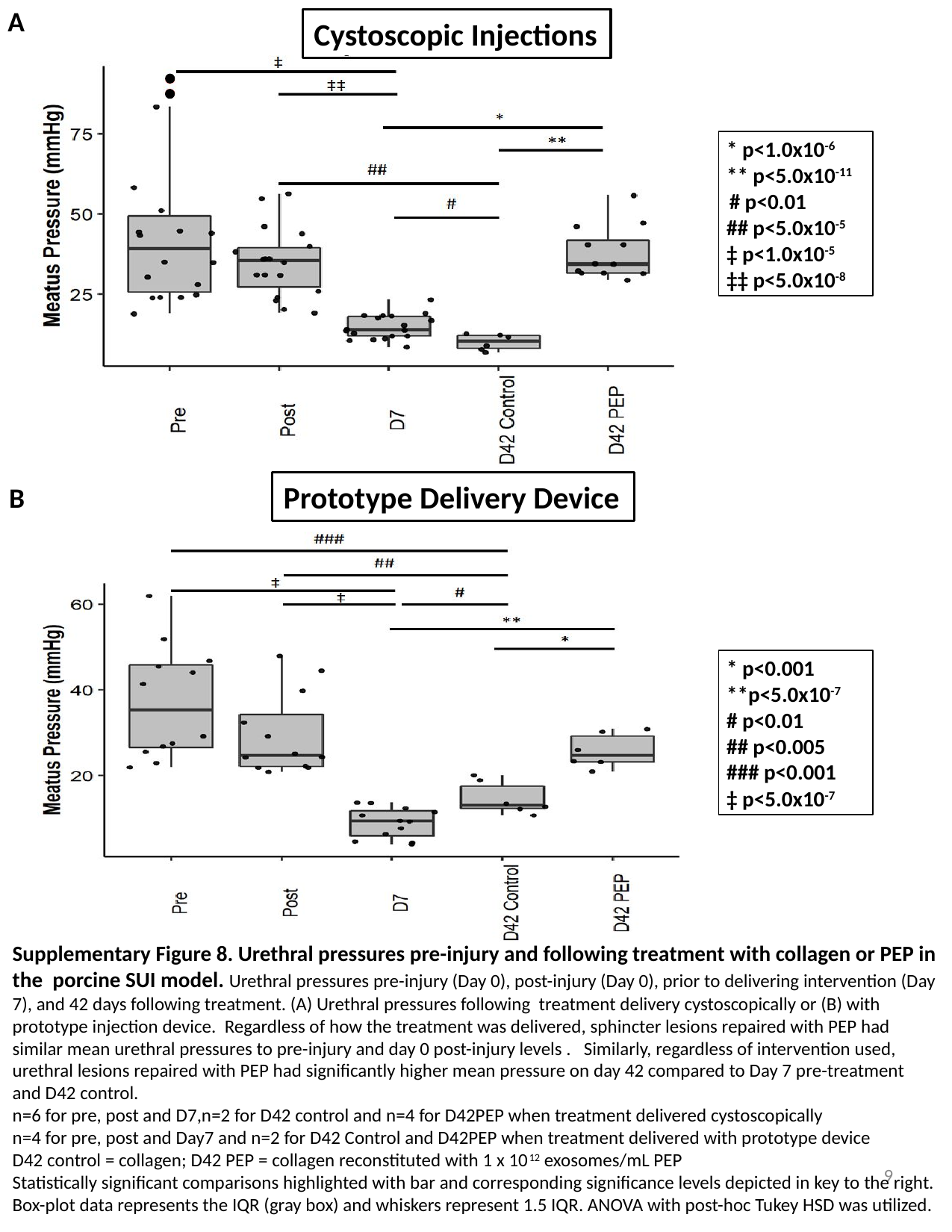

A
Cystoscopic Injections
* p<1.0x10^-6^
** p<5.0x10^-11^
 # p<0.01
## p<5.0x10^-5^
‡ p<1.0x10^-5^
‡‡ p<5.0x10^-8^
Prototype Delivery Device
B
* p<0.001
**p<5.0x10^-7^
# p<0.01
## p<0.005
### p<0.001
‡ p<5.0x10^-7^
Supplementary Figure 8. Urethral pressures pre-injury and following treatment with collagen or PEP in the porcine SUI model. Urethral pressures pre-injury (Day 0), post-injury (Day 0), prior to delivering intervention (Day 7), and 42 days following treatment. (A) Urethral pressures following treatment delivery cystoscopically or (B) with prototype injection device. Regardless of how the treatment was delivered, sphincter lesions repaired with PEP had similar mean urethral pressures to pre-injury and day 0 post-injury levels . Similarly, regardless of intervention used, urethral lesions repaired with PEP had significantly higher mean pressure on day 42 compared to Day 7 pre-treatment and D42 control.
n=6 for pre, post and D7,n=2 for D42 control and n=4 for D42PEP when treatment delivered cystoscopically
n=4 for pre, post and Day7 and n=2 for D42 Control and D42PEP when treatment delivered with prototype device
D42 control = collagen; D42 PEP = collagen reconstituted with 1 x 10^12^ exosomes/mL PEP
Statistically significant comparisons highlighted with bar and corresponding significance levels depicted in key to the right.
Box-plot data represents the IQR (gray box) and whiskers represent 1.5 IQR. ANOVA with post-hoc Tukey HSD was utilized.
9

## Slide 10
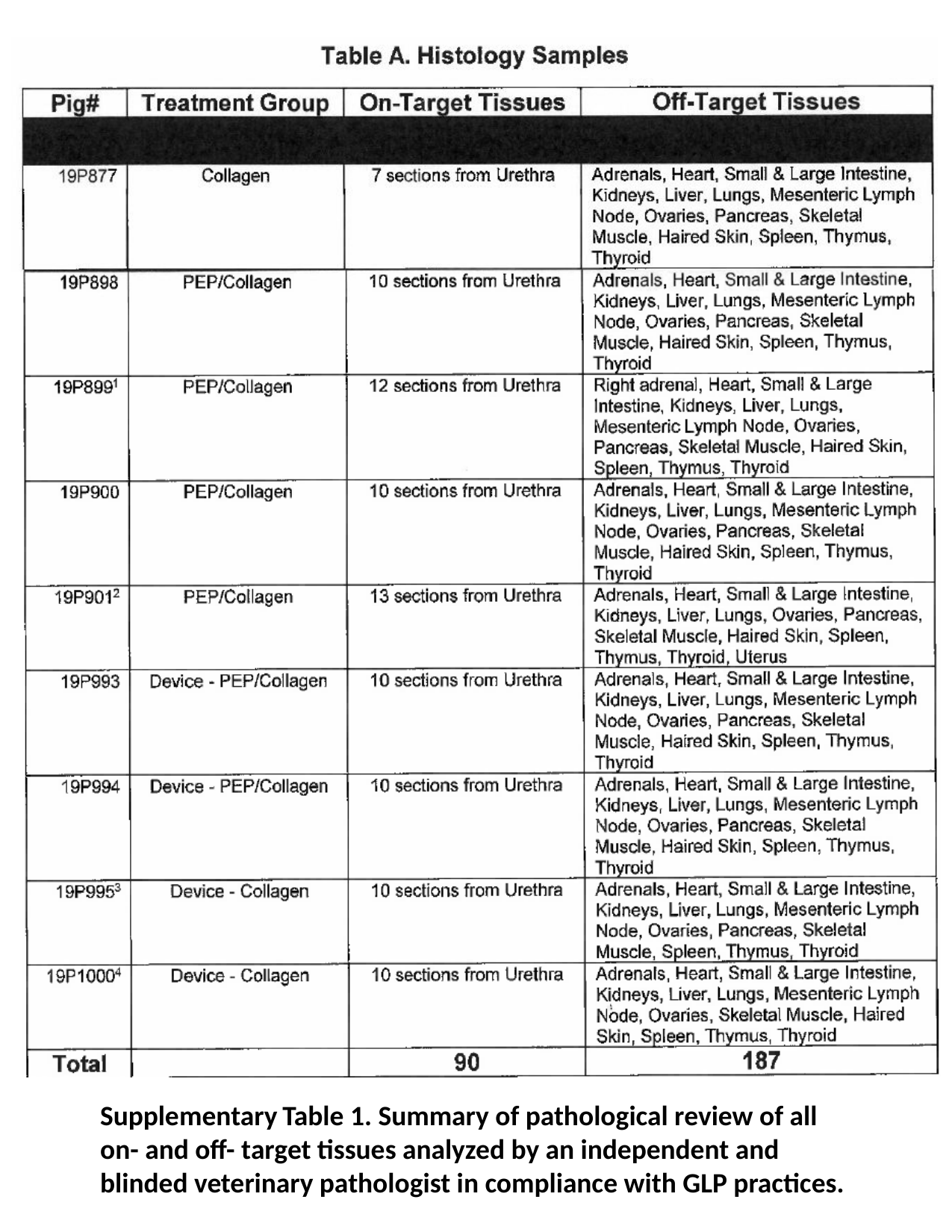

Supplementary Table 1. Summary of pathological review of all on- and off- target tissues analyzed by an independent and blinded veterinary pathologist in compliance with GLP practices.

## Slide 11
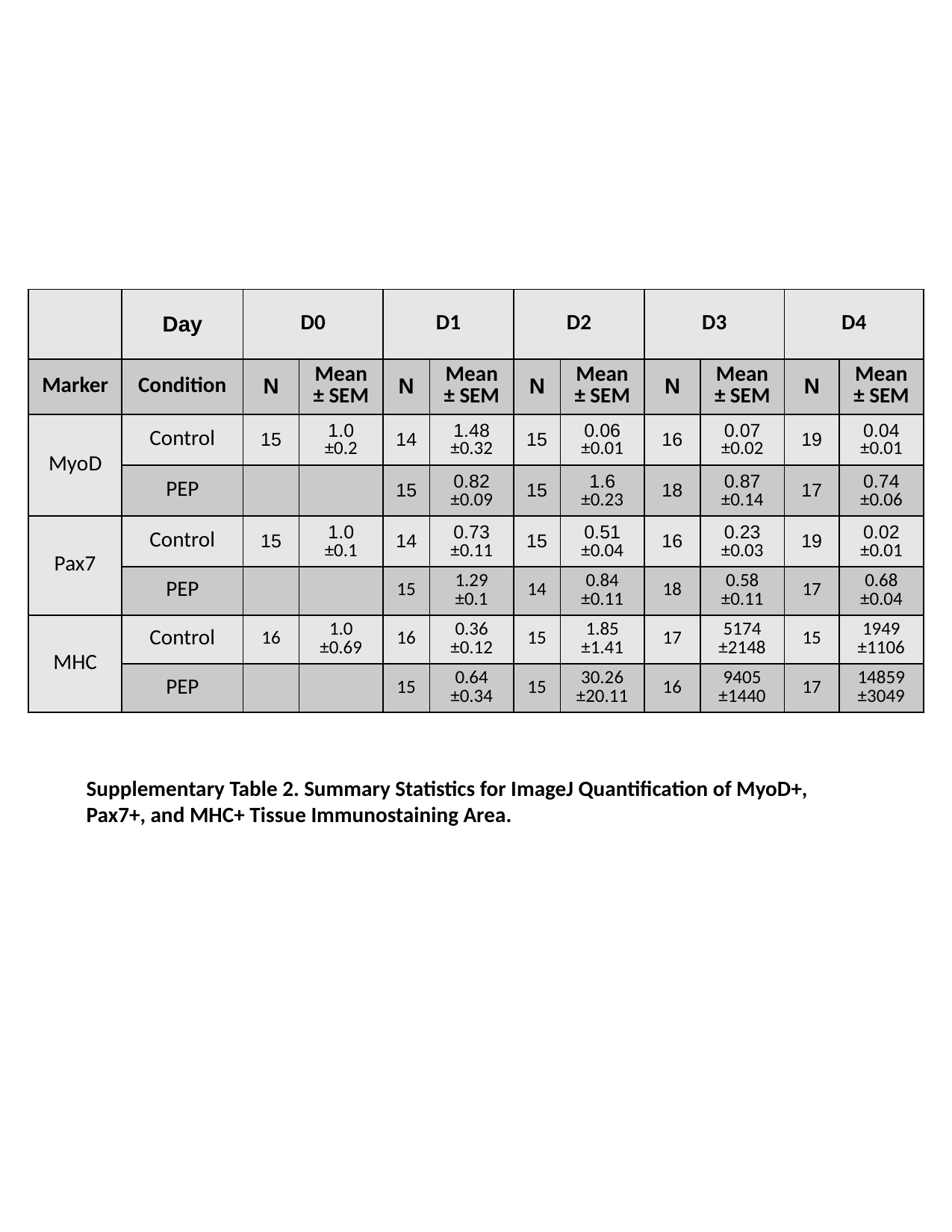

| | Day | D0 | | D1 | | D2 | | D3 | | D4 | |
| --- | --- | --- | --- | --- | --- | --- | --- | --- | --- | --- | --- |
| Marker | Condition | N | Mean ± SEM | N | Mean ± SEM | N | Mean ± SEM | N | Mean ± SEM | N | Mean ± SEM |
| MyoD | Control | 15 | 1.0 ±0.2 | 14 | 1.48 ±0.32 | 15 | 0.06 ±0.01 | 16 | 0.07 ±0.02 | 19 | 0.04 ±0.01 |
| | PEP | | | 15 | 0.82 ±0.09 | 15 | 1.6 ±0.23 | 18 | 0.87 ±0.14 | 17 | 0.74 ±0.06 |
| Pax7 | Control | 15 | 1.0 ±0.1 | 14 | 0.73 ±0.11 | 15 | 0.51 ±0.04 | 16 | 0.23 ±0.03 | 19 | 0.02 ±0.01 |
| | PEP | | | 15 | 1.29 ±0.1 | 14 | 0.84 ±0.11 | 18 | 0.58 ±0.11 | 17 | 0.68 ±0.04 |
| MHC | Control | 16 | 1.0 ±0.69 | 16 | 0.36 ±0.12 | 15 | 1.85 ±1.41 | 17 | 5174 ±2148 | 15 | 1949 ±1106 |
| | PEP | | | 15 | 0.64 ±0.34 | 15 | 30.26 ±20.11 | 16 | 9405 ±1440 | 17 | 14859 ±3049 |
Supplementary Table 2. Summary Statistics for ImageJ Quantification of MyoD+, Pax7+, and MHC+ Tissue Immunostaining Area.

## Slide 12
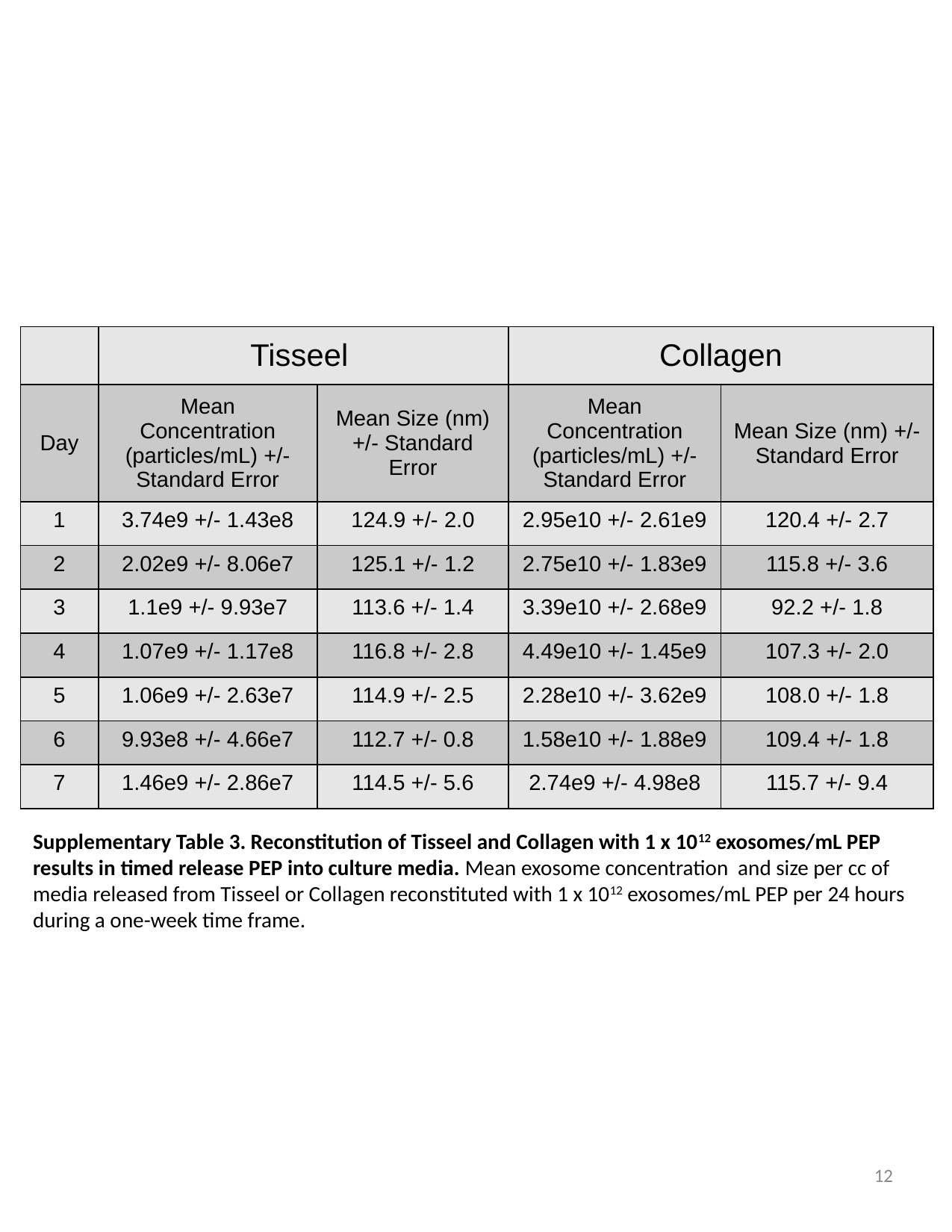

| | Tisseel | | Collagen | |
| --- | --- | --- | --- | --- |
| Day | Mean Concentration (particles/mL) +/- Standard Error | Mean Size (nm) +/- Standard Error | Mean Concentration (particles/mL) +/- Standard Error | Mean Size (nm) +/- Standard Error |
| 1 | 3.74e9 +/- 1.43e8 | 124.9 +/- 2.0 | 2.95e10 +/- 2.61e9 | 120.4 +/- 2.7 |
| 2 | 2.02e9 +/- 8.06e7 | 125.1 +/- 1.2 | 2.75e10 +/- 1.83e9 | 115.8 +/- 3.6 |
| 3 | 1.1e9 +/- 9.93e7 | 113.6 +/- 1.4 | 3.39e10 +/- 2.68e9 | 92.2 +/- 1.8 |
| 4 | 1.07e9 +/- 1.17e8 | 116.8 +/- 2.8 | 4.49e10 +/- 1.45e9 | 107.3 +/- 2.0 |
| 5 | 1.06e9 +/- 2.63e7 | 114.9 +/- 2.5 | 2.28e10 +/- 3.62e9 | 108.0 +/- 1.8 |
| 6 | 9.93e8 +/- 4.66e7 | 112.7 +/- 0.8 | 1.58e10 +/- 1.88e9 | 109.4 +/- 1.8 |
| 7 | 1.46e9 +/- 2.86e7 | 114.5 +/- 5.6 | 2.74e9 +/- 4.98e8 | 115.7 +/- 9.4 |
Supplementary Table 3. Reconstitution of Tisseel and Collagen with 1 x 10^12^ exosomes/mL PEP results in timed release PEP into culture media. Mean exosome concentration and size per cc of media released from Tisseel or Collagen reconstituted with 1 x 10^12^ exosomes/mL PEP per 24 hours during a one-week time frame.
12

## Slide 13
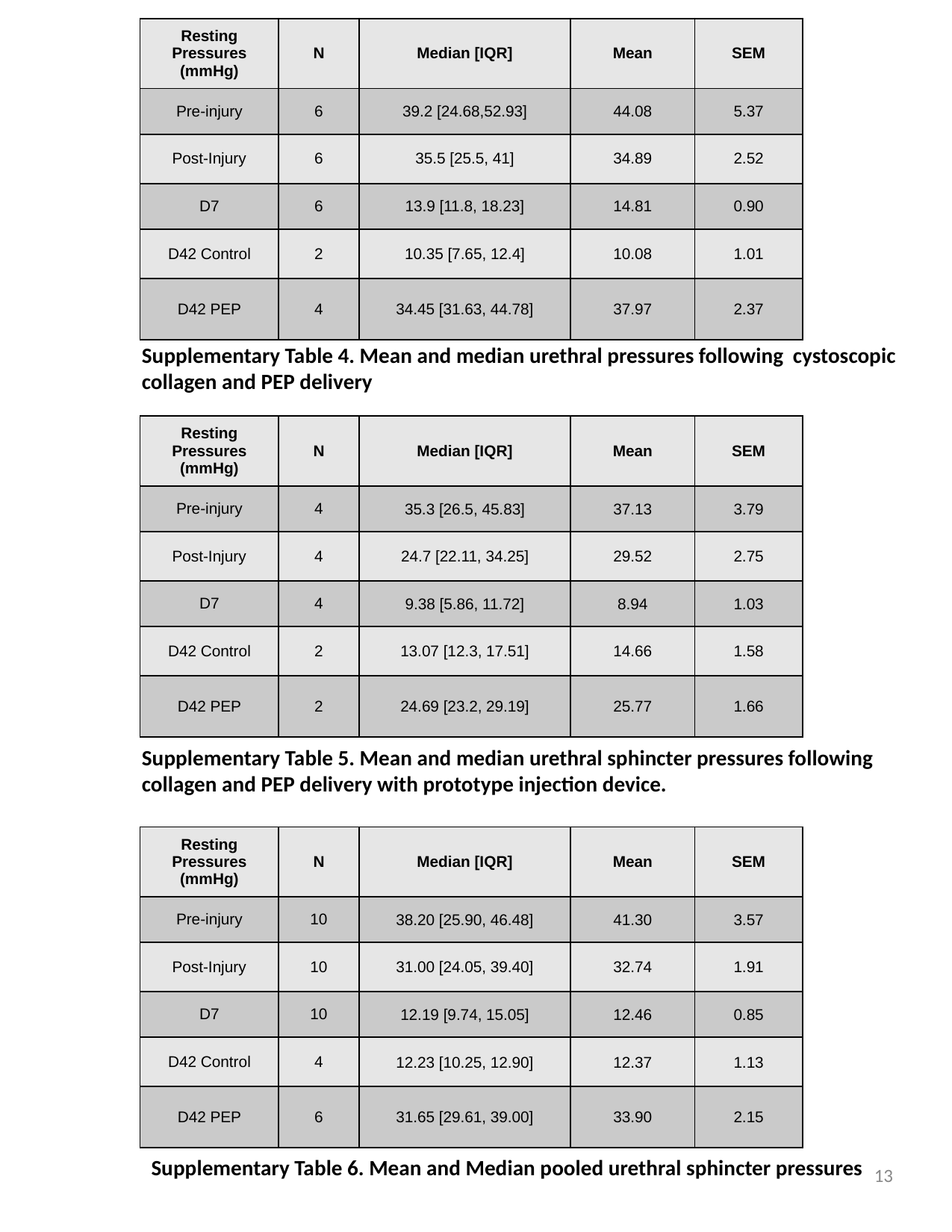

| Resting Pressures (mmHg) | N | Median [IQR] | Mean | SEM |
| --- | --- | --- | --- | --- |
| Pre-injury | 6 | 39.2 [24.68,52.93] | 44.08 | 5.37 |
| Post-Injury | 6 | 35.5 [25.5, 41] | 34.89 | 2.52 |
| D7 | 6 | 13.9 [11.8, 18.23] | 14.81 | 0.90 |
| D42 Control | 2 | 10.35 [7.65, 12.4] | 10.08 | 1.01 |
| D42 PEP | 4 | 34.45 [31.63, 44.78] | 37.97 | 2.37 |
Supplementary Table 4. Mean and median urethral pressures following cystoscopic collagen and PEP delivery
| Resting Pressures (mmHg) | N | Median [IQR] | Mean | SEM |
| --- | --- | --- | --- | --- |
| Pre-injury | 4 | 35.3 [26.5, 45.83] | 37.13 | 3.79 |
| Post-Injury | 4 | 24.7 [22.11, 34.25] | 29.52 | 2.75 |
| D7 | 4 | 9.38 [5.86, 11.72] | 8.94 | 1.03 |
| D42 Control | 2 | 13.07 [12.3, 17.51] | 14.66 | 1.58 |
| D42 PEP | 2 | 24.69 [23.2, 29.19] | 25.77 | 1.66 |
Supplementary Table 5. Mean and median urethral sphincter pressures following collagen and PEP delivery with prototype injection device.
| Resting Pressures (mmHg) | N | Median [IQR] | Mean | SEM |
| --- | --- | --- | --- | --- |
| Pre-injury | 10 | 38.20 [25.90, 46.48] | 41.30 | 3.57 |
| Post-Injury | 10 | 31.00 [24.05, 39.40] | 32.74 | 1.91 |
| D7 | 10 | 12.19 [9.74, 15.05] | 12.46 | 0.85 |
| D42 Control | 4 | 12.23 [10.25, 12.90] | 12.37 | 1.13 |
| D42 PEP | 6 | 31.65 [29.61, 39.00] | 33.90 | 2.15 |
13
Supplementary Table 6. Mean and Median pooled urethral sphincter pressures

## Slide 14
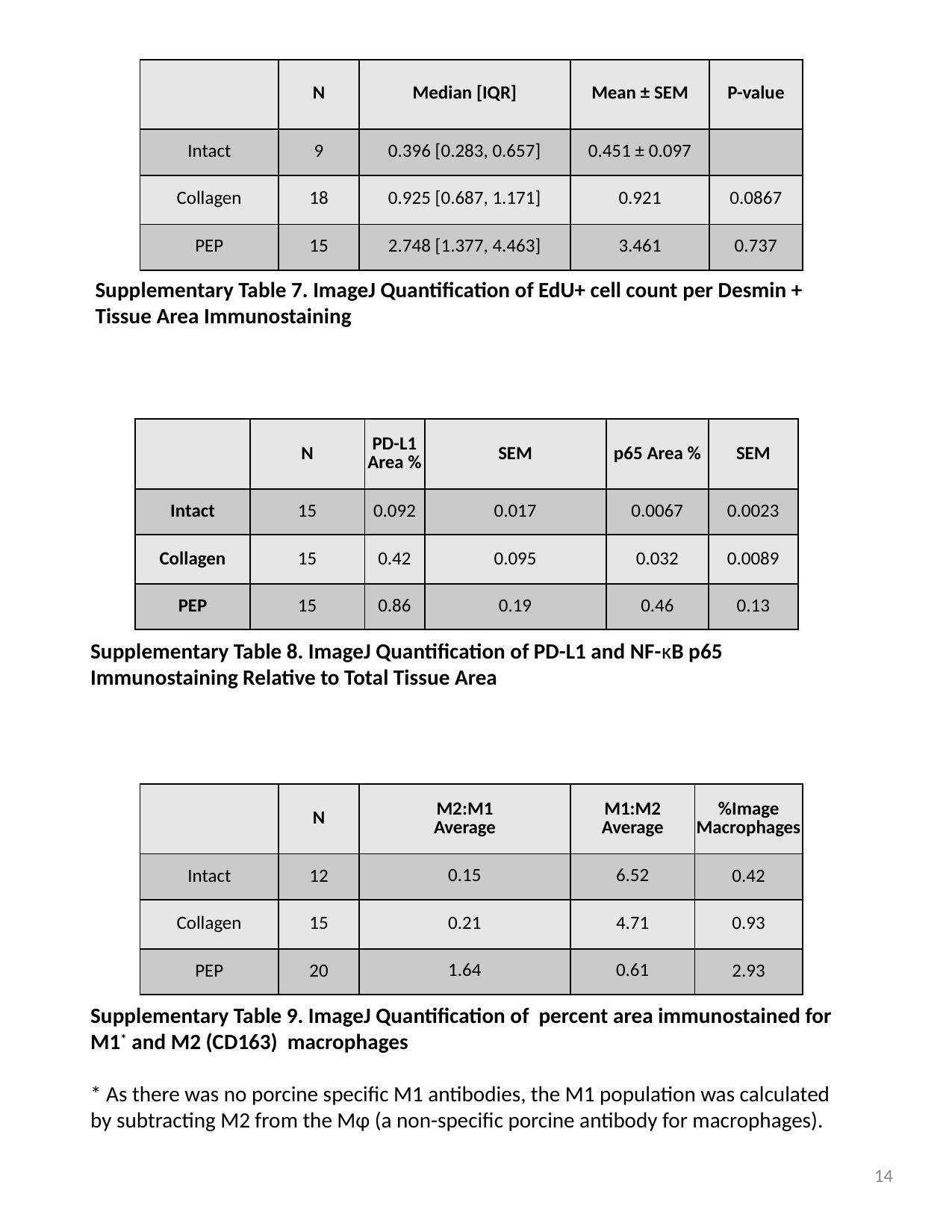

| | N | Median [IQR] | Mean ± SEM | P-value |
| --- | --- | --- | --- | --- |
| Intact | 9 | 0.396 [0.283, 0.657] | 0.451 ± 0.097 | |
| Collagen | 18 | 0.925 [0.687, 1.171] | 0.921 | 0.0867 |
| PEP | 15 | 2.748 [1.377, 4.463] | 3.461 | 0.737 |
Supplementary Table 7. ImageJ Quantification of EdU+ cell count per Desmin + Tissue Area Immunostaining
| | N | PD-L1 Area % | SEM | p65 Area % | SEM |
| --- | --- | --- | --- | --- | --- |
| Intact | 15 | 0.092 | 0.017 | 0.0067 | 0.0023 |
| Collagen | 15 | 0.42 | 0.095 | 0.032 | 0.0089 |
| PEP | 15 | 0.86 | 0.19 | 0.46 | 0.13 |
Supplementary Table 8. ImageJ Quantification of PD-L1 and NF-κB p65 Immunostaining Relative to Total Tissue Area
| | N | M2:M1 Average | M1:M2 Average | %Image Macrophages |
| --- | --- | --- | --- | --- |
| Intact | 12 | 0.15 | 6.52 | 0.42 |
| Collagen | 15 | 0.21 | 4.71 | 0.93 |
| PEP | 20 | 1.64 | 0.61 | 2.93 |
Supplementary Table 9. ImageJ Quantification of percent area immunostained for M1* and M2 (CD163) macrophages
* As there was no porcine specific M1 antibodies, the M1 population was calculated by subtracting M2 from the Mφ (a non-specific porcine antibody for macrophages).
14

## Slide 15
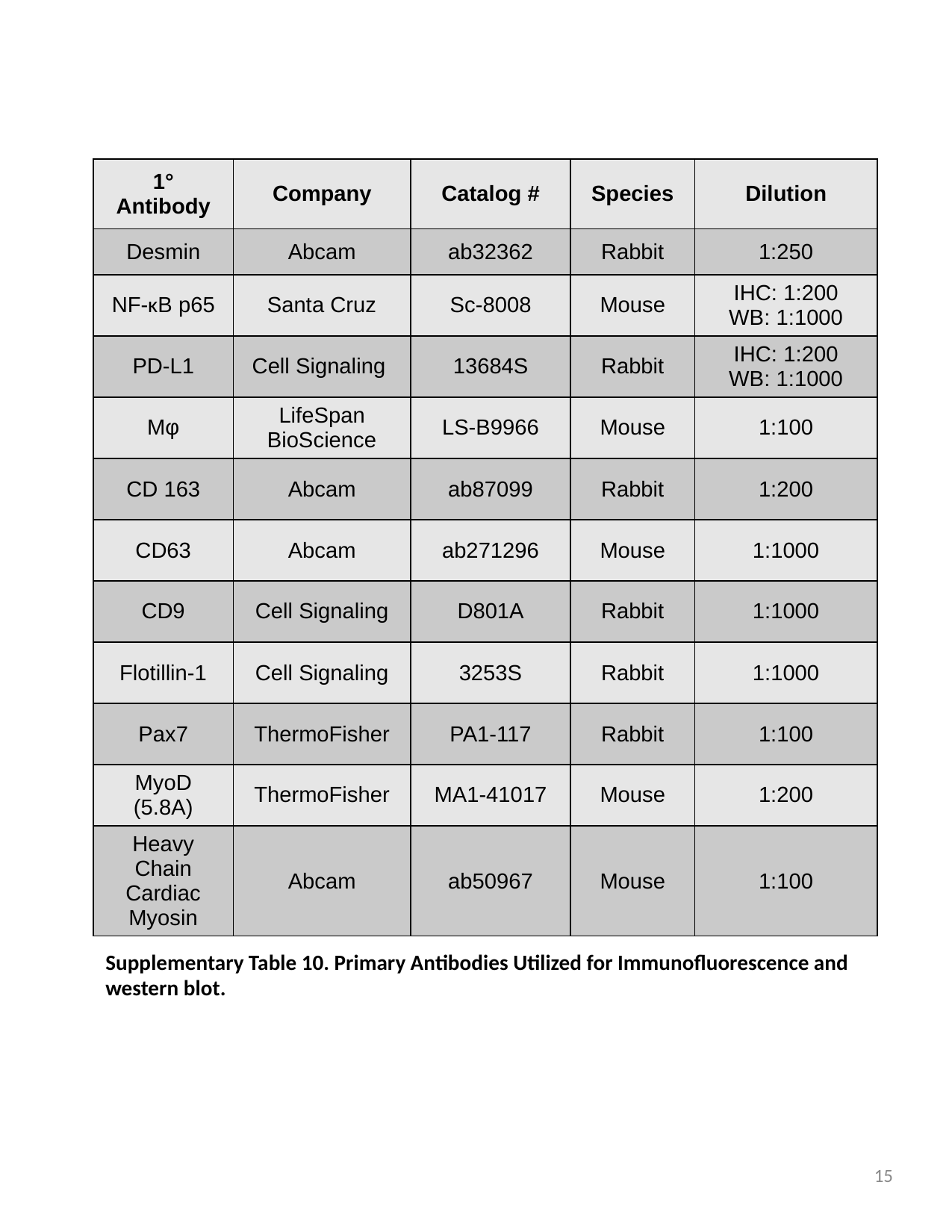

| 1° Antibody | Company | Catalog # | Species | Dilution |
| --- | --- | --- | --- | --- |
| Desmin | Abcam | ab32362 | Rabbit | 1:250 |
| NF-κB p65 | Santa Cruz | Sc-8008 | Mouse | IHC: 1:200 WB: 1:1000 |
| PD-L1 | Cell Signaling | 13684S | Rabbit | IHC: 1:200 WB: 1:1000 |
| Mφ | LifeSpan BioScience | LS-B9966 | Mouse | 1:100 |
| CD 163 | Abcam | ab87099 | Rabbit | 1:200 |
| CD63 | Abcam | ab271296 | Mouse | 1:1000 |
| CD9 | Cell Signaling | D801A | Rabbit | 1:1000 |
| Flotillin-1 | Cell Signaling | 3253S | Rabbit | 1:1000 |
| Pax7 | ThermoFisher | PA1-117 | Rabbit | 1:100 |
| MyoD (5.8A) | ThermoFisher | MA1-41017 | Mouse | 1:200 |
| Heavy Chain Cardiac Myosin | Abcam | ab50967 | Mouse | 1:100 |
Supplementary Table 10. Primary Antibodies Utilized for Immunofluorescence and western blot.
15

## Slide 16
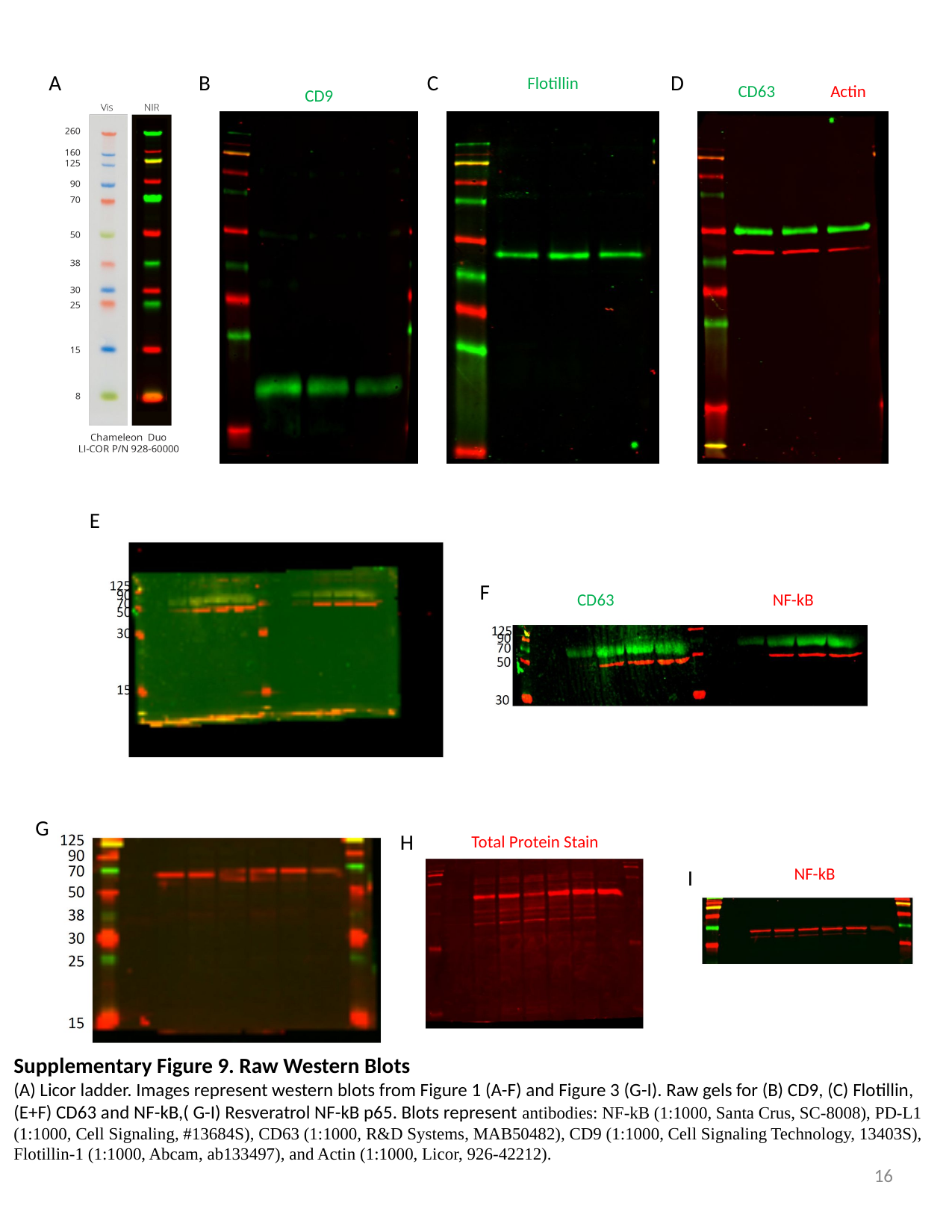

A
B
C
D
Flotillin
CD63
Actin
CD9
E
F
CD63
NF-kB
G
H
Total Protein Stain
I
NF-kB
Supplementary Figure 9. Raw Western Blots
(A) Licor ladder. Images represent western blots from Figure 1 (A-F) and Figure 3 (G-I). Raw gels for (B) CD9, (C) Flotillin, (E+F) CD63 and NF-kB,( G-I) Resveratrol NF-kB p65. Blots represent antibodies: NF-kB (1:1000, Santa Crus, SC-8008), PD-L1 (1:1000, Cell Signaling, #13684S), CD63 (1:1000, R&D Systems, MAB50482), CD9 (1:1000, Cell Signaling Technology, 13403S), Flotillin-1 (1:1000, Abcam, ab133497), and Actin (1:1000, Licor, 926-42212).
16

## Slide 17
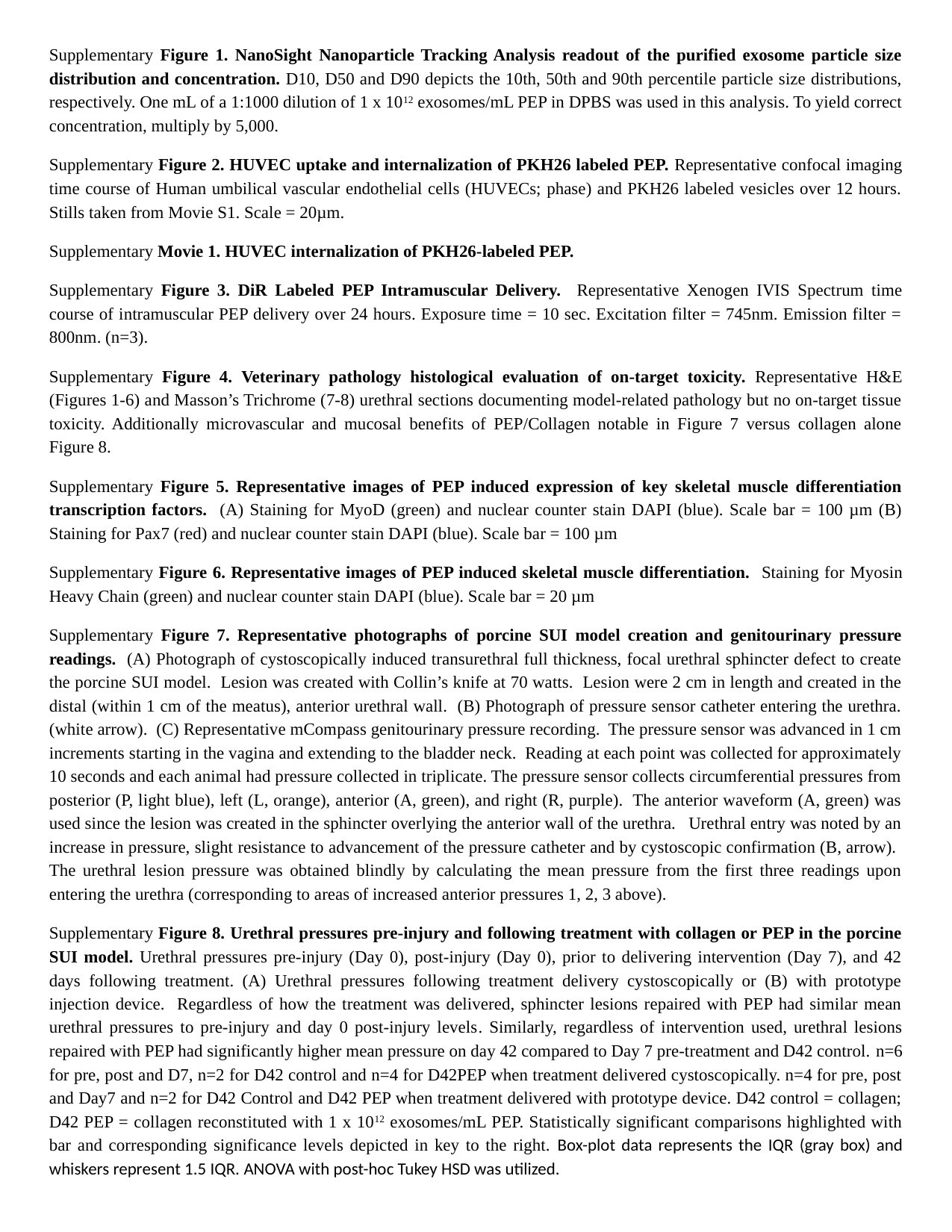

Supplementary Figure 1. NanoSight Nanoparticle Tracking Analysis readout of the purified exosome particle size distribution and concentration. D10, D50 and D90 depicts the 10th, 50th and 90th percentile particle size distributions, respectively. One mL of a 1:1000 dilution of 1 x 10^12^ exosomes/mL PEP in DPBS was used in this analysis. To yield correct concentration, multiply by 5,000.
Supplementary Figure 2. HUVEC uptake and internalization of PKH26 labeled PEP. Representative confocal imaging time course of Human umbilical vascular endothelial cells (HUVECs; phase) and PKH26 labeled vesicles over 12 hours. Stills taken from Movie S1. Scale = 20µm.
Supplementary Movie 1. HUVEC internalization of PKH26-labeled PEP.
Supplementary Figure 3. DiR Labeled PEP Intramuscular Delivery. Representative Xenogen IVIS Spectrum time course of intramuscular PEP delivery over 24 hours. Exposure time = 10 sec. Excitation filter = 745nm. Emission filter = 800nm. (n=3).
Supplementary Figure 4. Veterinary pathology histological evaluation of on-target toxicity. Representative H&E (Figures 1-6) and Masson’s Trichrome (7-8) urethral sections documenting model-related pathology but no on-target tissue toxicity. Additionally microvascular and mucosal benefits of PEP/Collagen notable in Figure 7 versus collagen alone Figure 8.
Supplementary Figure 5. Representative images of PEP induced expression of key skeletal muscle differentiation transcription factors. (A) Staining for MyoD (green) and nuclear counter stain DAPI (blue). Scale bar = 100 µm (B) Staining for Pax7 (red) and nuclear counter stain DAPI (blue). Scale bar = 100 µm
Supplementary Figure 6. Representative images of PEP induced skeletal muscle differentiation. Staining for Myosin Heavy Chain (green) and nuclear counter stain DAPI (blue). Scale bar = 20 µm
Supplementary Figure 7. Representative photographs of porcine SUI model creation and genitourinary pressure readings. (A) Photograph of cystoscopically induced transurethral full thickness, focal urethral sphincter defect to create the porcine SUI model. Lesion was created with Collin’s knife at 70 watts. Lesion were 2 cm in length and created in the distal (within 1 cm of the meatus), anterior urethral wall. (B) Photograph of pressure sensor catheter entering the urethra. (white arrow). (C) Representative mCompass genitourinary pressure recording. The pressure sensor was advanced in 1 cm increments starting in the vagina and extending to the bladder neck. Reading at each point was collected for approximately 10 seconds and each animal had pressure collected in triplicate. The pressure sensor collects circumferential pressures from posterior (P, light blue), left (L, orange), anterior (A, green), and right (R, purple). The anterior waveform (A, green) was used since the lesion was created in the sphincter overlying the anterior wall of the urethra. Urethral entry was noted by an increase in pressure, slight resistance to advancement of the pressure catheter and by cystoscopic confirmation (B, arrow). The urethral lesion pressure was obtained blindly by calculating the mean pressure from the first three readings upon entering the urethra (corresponding to areas of increased anterior pressures 1, 2, 3 above).
Supplementary Figure 8. Urethral pressures pre-injury and following treatment with collagen or PEP in the porcine SUI model. Urethral pressures pre-injury (Day 0), post-injury (Day 0), prior to delivering intervention (Day 7), and 42 days following treatment. (A) Urethral pressures following treatment delivery cystoscopically or (B) with prototype injection device. Regardless of how the treatment was delivered, sphincter lesions repaired with PEP had similar mean urethral pressures to pre-injury and day 0 post-injury levels. Similarly, regardless of intervention used, urethral lesions repaired with PEP had significantly higher mean pressure on day 42 compared to Day 7 pre-treatment and D42 control. n=6 for pre, post and D7, n=2 for D42 control and n=4 for D42PEP when treatment delivered cystoscopically. n=4 for pre, post and Day7 and n=2 for D42 Control and D42 PEP when treatment delivered with prototype device. D42 control = collagen; D42 PEP = collagen reconstituted with 1 x 10^12^ exosomes/mL PEP. Statistically significant comparisons highlighted with bar and corresponding significance levels depicted in key to the right. Box-plot data represents the IQR (gray box) and whiskers represent 1.5 IQR. ANOVA with post-hoc Tukey HSD was utilized.

## Slide 18
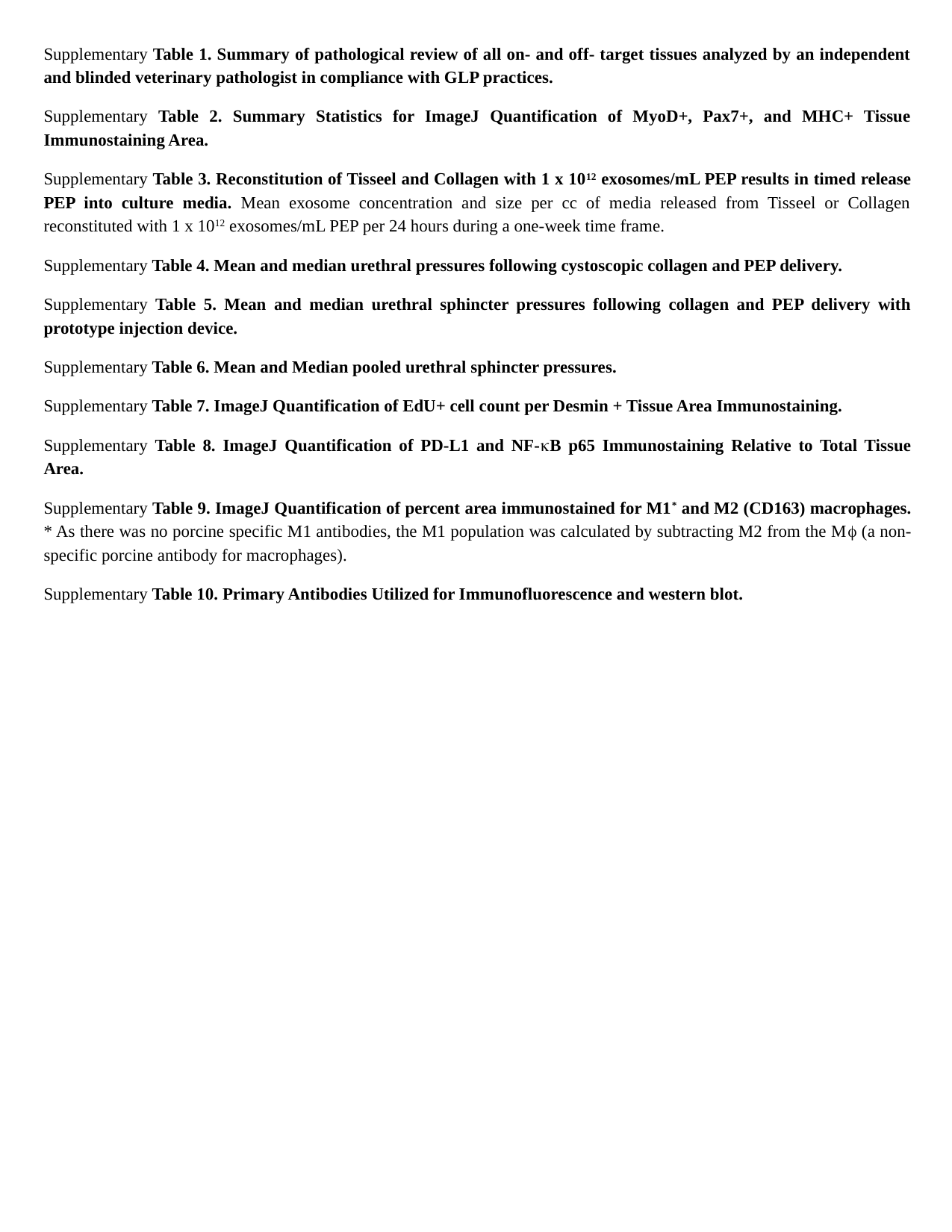

Supplementary Table 1. Summary of pathological review of all on- and off- target tissues analyzed by an independent and blinded veterinary pathologist in compliance with GLP practices.
Supplementary Table 2. Summary Statistics for ImageJ Quantification of MyoD+, Pax7+, and MHC+ Tissue Immunostaining Area.
Supplementary Table 3. Reconstitution of Tisseel and Collagen with 1 x 10^12^ exosomes/mL PEP results in timed release PEP into culture media. Mean exosome concentration and size per cc of media released from Tisseel or Collagen reconstituted with 1 x 10^12^ exosomes/mL PEP per 24 hours during a one-week time frame.
Supplementary Table 4. Mean and median urethral pressures following cystoscopic collagen and PEP delivery.
Supplementary Table 5. Mean and median urethral sphincter pressures following collagen and PEP delivery with prototype injection device.
Supplementary Table 6. Mean and Median pooled urethral sphincter pressures.
Supplementary Table 7. ImageJ Quantification of EdU+ cell count per Desmin + Tissue Area Immunostaining.
Supplementary Table 8. ImageJ Quantification of PD-L1 and NF-κB p65 Immunostaining Relative to Total Tissue Area.
Supplementary Table 9. ImageJ Quantification of percent area immunostained for M1* and M2 (CD163) macrophages. * As there was no porcine specific M1 antibodies, the M1 population was calculated by subtracting M2 from the Mφ (a non-specific porcine antibody for macrophages).
Supplementary Table 10. Primary Antibodies Utilized for Immunofluorescence and western blot.
